# Trends in all-cause mortality among adults with diagnosed type 2 diabetes in West Malaysia: 2010 – 2019

**DOI:** 10.1016/j.diabres.2023.110944

**Published:** 2023-11

**Authors:** Lee-Ling Lim, Alia Abdul Aziz, Helen Dakin, John Buckell, Yuan-Liang Woon, Laurence Roope, Arunah Chandran, Feisul I. Mustapha, Edward W. Gregg, Philip M. Clarke

**Affiliations:** a Department of Medicine, Faculty of Medicine, University of Malaya, Kuala Lumpur, Malaysia; b Department of Medicine and Therapeutics, The Chinese University of Hong Kong, Hong Kong SAR, China; c Asia Diabetes Foundation, Hong Kong SAR, China; d Health Economics Research Centre, Nuffield Department of Public Health, University of Oxford, Oxford, United Kingdom; e Centre for Clinical Epidemiology, Institute for Clinical Research, National Institute of Health, Selangor, Malaysia; f Non-communicable Disease Section, Disease Control Division, Ministry of Health, Putrajaya, Malaysia; g Department of Epidemiology and Biostatistics, School of Public Health, Imperial College London, London, United Kingdom

**Keywords:** Cardiovascular disease, Diabetes mellitus, Epidemiology, Ethnicity, Health disparity, Middle-income country

## Abstract

**Aims:**

We determined 10-year all-cause mortality trends in diagnosed type 2 diabetes (T2D) population in West Malaysia, a middle-income country in the Western-Pacific region.

**Methods:**

One million T2D people aged 40–79 registered in the National Diabetes Registry (2009–2018) were linked to death records (censored on 31 December 2019). Standardized absolute mortality rates and standardized mortality ratios (SMRs) were estimated relative to the Malaysian general population, and standardized to the 2019 registry population with respect to sex, age group, and disease duration.

**Results:**

Overall all-cause standardized mortality rates were unchanged in both sexes. Rates increased in males aged 40–49 (annual average percent change [AAPC]: 2.46 % [95 % CI 0.42 %, 4.55 %]) and 50–59 (AAPC: 1.91 % [95 % CI 0.73 %, 3.10 %]), and females aged 40–49 (AAPC: 3.39 % [95 % CI 1.32 %, 5.50 %]). In both sexes, rates increased among those with 1) > 15 years disease duration, 2) prior cardiovascular disease, and 3) Bumiputera (Malay/native) ethnicity. The overall SMR was 1.83 (95 % CI 1.80, 1.86) for males and 1.85 (95 % CI 1.82, 1.89) for females, being higher in younger age groups and showed an increasing trend in those with either > 15 years disease duration or prior cardiovascular disease.

**Conclusions:**

Mortality trends worsened in certain T2D population in Malaysia.

## 1 Introduction

Diabetes is an enormous global health problem in all regions of the world, now affecting 9 % of adults [1], [2]. It can lead to multimorbidity and reduced life expectancy with a loss of 5–7 years of life from the age of 40 years, primarily derived from data published by the high-income countries [3], [4], [5]. Several studies have shown that rates of mortality among people with diagnosed diabetes have decreased, often at a faster rate than in populations without diabetes [3], [6], [7], [8]. However, to date, all studies of trends in all-cause mortality have been conducted in high-income countries [3], [9]. Whether populations with diabetes in low- and middle-income countries (LMICs) are experiencing similar improvements is unknown. This represents a major international disparity in health data [10], especially given that 80 % of people with diagnosed diabetes live in LMICs [2].

International comparisons of diabetes health services have shown that LMICs have considerably poorer management of glycemic control and cardiovascular risk factors, leading to higher cardiovascular mortality [11], [12], [13], [14], [15]. These differences raise the question of whether the temporal trends in all-cause mortality for diabetes also differ between regions and whether improvements in care and reductions in mortality are also seen in LMICs.

Amongst LMICs, Southeast Asia is a particular area of concern due to rapid increases in diabetes and obesity with suboptimal health system performance [14], [16]. Alongside national estimates, there is a need to be able to track the burden of diabetes at the national level. Several countries have developed diabetes registries, but these are largely based in high-income countries. In Malaysia, the National Diabetes Registry (NDR) was established in 2009 by the Ministry of Health (MOH) to monitor the clinical outcomes of people with diabetes that are managed in publicly funded primary care clinics [17]. This provides an opportunity to examine whether people with diabetes are experiencing the reductions in mortality rates observed in high-income countries.

Using the Malaysian NDR, we aimed to examine the trends in all-cause mortality among the Malaysian population with diagnosed type 2 diabetes, and to determine whether they differed from those of the general population. We also aimed to examine the variations in mortality rates and trends according to sex, age group, and ethnicity within Malaysia.

## 2 Methods

### 2.1 Data sources and Research design

Malaysia is an upper middle-income country in Southeast Asia with 33.4 million population [18]. The country has 13 states and three federal territories, separated into West (27.2 million population; 81.4 %) and East Malaysia (6.2 million population; 18.6 %) [18]. West Malaysia consists of 11 out of 13 states and two out of three federal territories including the national capital of Kuala Lumpur. West Malaysia is further divided into four regions namely Central, East Coast, Northern, and Southern regions. Malaysia is a multi-ethnic country with three main ethnicities namely Bumiputera (a term used to describe Malays and other native people; 69.9 %), Chinese (22.8 %), and Indians (6.7 %) [18]. People who do not fall under the three main ethnic groups are classified as “Others” (0.6 %) [18].

In Malaysia, 74 % of people with diagnosed diabetes receive care at publicly-funded primary care clinics and 68 % of them who have at least one outpatient clinic visit within one year of the data collection period of NDR are registered [19], [20]. In the NDR, diabetes is diagnosed based on 1) fasting plasma glucose ≥ 7.0 mmol/L after an 8-hour overnight fast, 2) 2-hour plasma glucose after a 75-gram oral glucose tolerance test ≥ 11.1 mmol/L, or 3) an HbA_1c_ of ≥ 45 mmol/mol (6.3 %) which was derived from a local community-based study and introduced in 2015 [21], [22]. This cut-off had a sensitivity of 42.5 % and a specificity of 97.4 % for diagnosing diabetes among Malaysians, whilst the corresponding values were 36.7 % and 98.1 % for HbA_1c_ ≥ 48 mmol/mol (6.5 %) which was the internationally recommended cut-off [22]. Those with either impaired fasting glucose, impaired glucose tolerance, or gestational diabetes mellitus are excluded.

The NDR transitioned from a paper-based case record form supported with an Excel-based stand-alone application in 2009 to a web-based data collection system on 1 January 2011, leading to a large increase in participants. The number of participating clinics increased from 644 (out of 879 publicly funded primary care clinics; 73 %) in 2011 to 830 (out of 1,027; 81 %) in 2019, with relatively stable representations from 13 states in Malaysia from 2012 (Table S1) [20]. Between 2009 and 2019, a total of 1,614,363 people with diabetes have been registered in the NDR, of whom 99 % have type 2 diabetes.

**Fig. 1:**
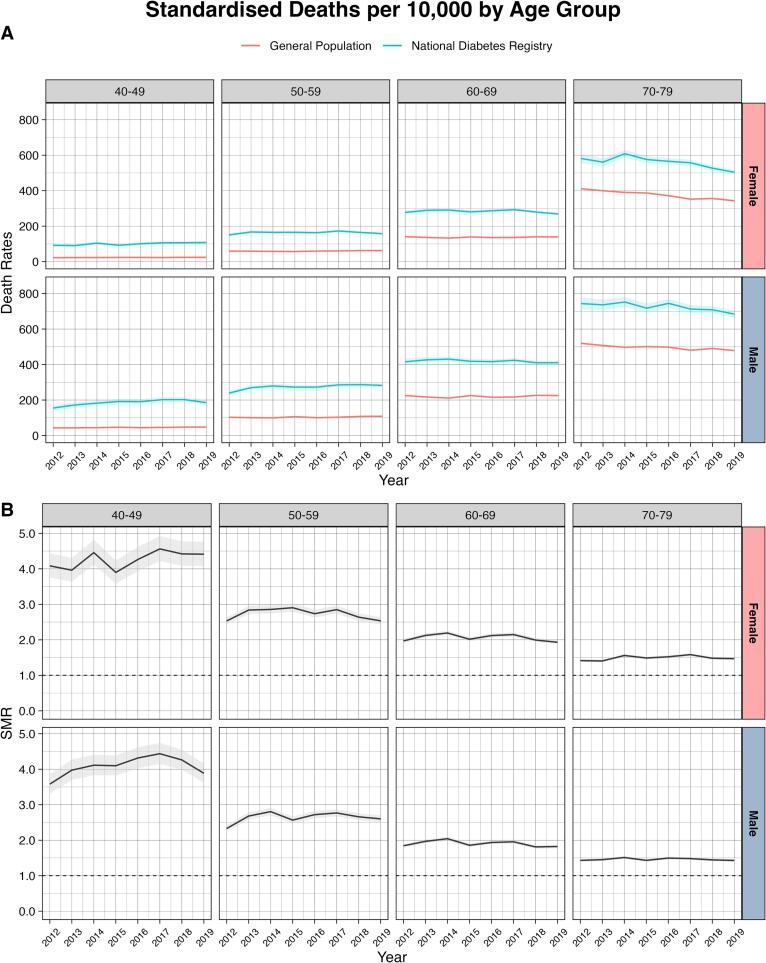
Trends in age-standardized all-cause mortality rates (A) and standardized mortality ratios (B) in people aged 40–79 years with diagnosed type 2 diabetes, stratified by age in that particular year (10-year bands) Footnotes: For mortality rates, confidence intervals were estimated using the Wald normal approximation interval and multiplied by 10,000. For standardized mortality ratio, confidence intervals were estimated using the Breslow and Day method.

Our analysis included all people registered in the NDR between 2 January 2009 and 31 December 2018 who met the following eligibility criteria:

1) Diagnosis of type 2 diabetes classified by the attending physician based on clinical presentations and laboratory tests.
2) Aged 40–79 years. We used 40 years as the cut-off of current age to reduce the number of people who might potentially have type 1 diabetes (n = 7,464 [0.6 %]) (Figure S1) [23]. In Malaysia, people with type 1 diabetes are generally managed in hospital settings and not in primary care. People aged 80 years and over were also excluded as Malaysian life tables only presented mortality by five-year age bands up to age 80, such that reliable SMRs could not be estimated for people aged 80 years and over.
3) Attending clinics in West Malaysia. In this analysis, we excluded 147,805 (11.9 %) people attending clinics in East Malaysia to minimize misclassification bias because death registration is not mandatory in East Malaysia (Fig. 1).

The NDR dataset was downloaded from the web-based system and matched to National Death Registry using unique identifiers (also known as a Malaysian identification card number). The unique identifiers of all citizens follow a 12-digit numbering system. Hence, in this analysis, missing sex was replaced using unique identifiers wherein the last digit represented male if odd numbers whilst female if even numbers [24]. All deaths occurring in West Malaysia are to be registered with the National Registration Department for issuing a death certificate [25]. All data were censored on 31 December 2019. Data on the cause of death was not available for this study because up to 34 % of deaths occur outside a healthcare facility and are not medically certified [26].

This study was approved by the MOH Medical Research and Ethics Committee (NMRR-19–3566-52314) and the University of Malaya Medical Centre, Malaysia (MREC ID 20191216–8082). There was a waiver of informed consent as this study involved secondary analysis of existing data.

### 2.2 Statistical analysis

We calculated age-specific all-cause mortality rates and standardized mortality ratios (SMRs) with 95 % confidence intervals (CI). We analyzed data on people with type 2 diabetes from their date of registration in the NDR on 2 January 2009 until either death or 31 December 2018. Since death registration is mandatory and emigration rates are low (1.6 % in 2018) [27] and not recorded in the NDR, we assumed that all people with type 2 diabetes not in the National Death Registry were still alive on 31 December 2019.

The population for each year of analysis comprised all people with type 2 diabetes who were registered by 31 December the previous year and were still alive on that date. The observed percentage mortality equaled the total number of people who died divided by the population size for each subgroup in each year. To ensure that trends were not confounded by changes in age structure, we used a direct standardization method [28] wherein we standardized each year to the registry population as of 2 January 2019 by age, sex, and duration of type 2 diabetes. Analyses subgrouping by age were standardized by the duration of type 2 diabetes and vice versa.

Mortality rates were estimated by dividing the standardized number of deaths by the standardized population size for that subgroup and year, multiplied by 10,000. Confidence intervals were estimated on the mortality rates using the Wald normal approximation interval and multiplied by 10,000. We estimated relative differences in mortality using SMRs [29]. Mortality rates for the general Malaysian population in each year of analysis (2009–2019) by age and sex were taken from abridged life tables [30]. All analysis of mortality rates and SMR involved matching each participant in the registry year at risk, age in that particular year, and sex to the relevant period in the life table. Subsequently, we calculated the SMR as the observed number of deaths within the standardized population in each year divided by the total number of expected deaths based on the life table. The Breslow and Day method was used to estimate confidence intervals [31]. To summarize overall trends, we plotted the SMRs over the calendar years.

To test the statistical significance of temporal trends in mortality rates across all years, we used fractional logistic regression to estimate how the standardized proportion of people dying varied with calendar year, with survey weights equal to the inverse of the variance. The annual average percent change (AAPC) in the odds of mortality was calculated from the odds ratio (OR) on calendar year estimated in the regression by $\mathrm{AAPC}=100\%(1-{\mathrm{OR}}_{\mathrm{year}})$.

We also performed sensitivity analyses wherein 1) the SMR for people with type 2 diabetes from clinics in the registry that were present in all years from 2012 to 2018 was compared with the SMR for people from all clinics within the same years, and 2) people with type 2 diabetes diagnosed at younger than 30 years of age were excluded. We stratified all analyses by 10-year bands for age in that particular year (40–49, 50–59, 60–69, and 70–79), sex (male and female), five-year bands for duration of type 2 diabetes (0–5, 5–10, 10–15, and > 15 years), prior history of cardiovascular disease (CVD; present and absent/unknown), ethnicity (Bumiputera, Chinese, Indian, and others), and geographic regions (Central, East Coast, Northern, and Southern region of West Malaysia). Overall life tables for males and females were used in all subgroup analyses except for subgroup analysis by ethnicity where ethnicity-specific life tables were used.

Fractional logistic regression was performed in Stata version 17 (College Station TX) and all other statistical analyses were performed using R version 4.1.0 [32]. All tests were two-sided with a 0.05 significance level.

## 3 Results

This study included 996,355 people aged 40–79 years with diagnosed type 2 diabetes from 830 publicly funded primary care clinics, who were registered in the NDR between 2 January 2009 and 31 December 2018 (Figure S1). Table 1 shows the characteristics of the total population at-risk for mortality in each year. There was a greater proportion of females ranging from 60.3 % in 2010 to 58.4 % in 2019. In males, the mean ± standard deviation (SD) age in that particular year was 59.2 ± 9.0 years in 2010 and 56.7 ± 9.2 years in 2019. The corresponding figures were 58.2 ± 9.2 years and 56.2 ± 9.2 years in females. In males, the median duration of type 2 diabetes declined from 4.3 (interquartile range [IQR] 2.0–7.6) years in 2010 to 1.7 (IQR: 0.4–5.2) years in 2019, whilst in females, there was a similar trend from 4.3 (IQR: 2.1–7.6) years to 2.0 (IQR: 0.4–5.7) years. Two-thirds of the study population were of Bumiputera ethnicity. In males, the proportion of prior CVD was 1,187 (6.6 %) in 2010 and 22,954 (6.8 %) in 2019, whilst the corresponding figures were 1,214 (4.5 %) and 19,291 (4.1 %) in females.

**Table 1:** Characteristics of people aged 40–79 years with diagnosed type 2 diabetes in the Malaysian National Diabetes Registry, stratified by sex.

	2010		2011		2012		2013		2014		2015		2016		2017		2018		2019	
	Male (n = 17,920)	Female (n = 27,256)	Male (n = 38,636)	Female (n = 61,932)	Male (n = 139,022)	Female (n = 205,082)	Male (n = 181,286)	Female (n = 262,368)	Male (n = 207,441)	Female (n = 298,124)	Male (n = 231,385)	Female (n = 330,774)	Male (n = 256,218)	Female (n = 364,375)	Male (n = 286,948)	Female (n = 404,796)	Male (n = 314,214)	Female (n = 441,949)	Male (n = 336,166)	Female (n = 472,552)
Age in that particular year, year	59.2 ± 9.0	58.2 ± 9.2	59.3 ± 9.0	58.1 ± 9.1	59.0 ± 9.1	58.4 ± 9.3	58.6 ± 9.2	58.0 ± 9.3	58.1 ± 9.2	57.6 ± 9.3	57.7 ± 9.2	57.2 ± 9.3	57.4 ± 9.2	56.9 ± 9.2	57.1 ± 9.2	56.7 ± 9.2	56.9 ± 9.2	56.5 ± 9.2	56.7 ± 9.2	56.2 ± 9.2
Age at diabetes onset, year	53.4 ± 9.6	52.6 ± 9.6	53.7 ± 9.5	52.6 ± 9.42	53.8 ± 9.6	53.1 ± 9.6	53.7 ± 9.6	53.1 ± 9.6	53.6 ± 9.6	53.0 ± 9.6	53.5 ± 9.6	52.9 ± 9.6	53.4 ± 9.6	52.8 ± 9.6	53.3 ± 9.6	52.7 ± 9.5	53.2 ± 9.6	52.6 ± 9.5	53.1 ± 9.6	52.5 ± 9.5
Duration of diabetes in that particular year, year^#^	4.3 (2.0–7.6)	4.3 (2.1–7.6)	4.2 (2.0–7.4)	4.4 (2.1–7.6)	3.9 (1.5–7.2)	4.1 (1.8–7.4)	3.4 (1.1–6.9)	3.8 (1.3–7.2)	2.9 (0.8–6.6)	3.2 (0.9–6.9)	2.5 (0.6–6.1)	2.9 (0.7–6.5)	2.1 (0.5–5.8)	2.6 (0.6–6.2)	2.0 (0.4–5.7)	2.3 (0.5–6.0)	1.9 (0.4–5.5)	2.1 (0.5–5.9)	1.7 (0.4–5.2)	2.0 (0.4–5.7)
**Region, n (%)**																				
Central	9,485 (52.9)	13,655 (50.1)	18,314 (47.4)	27,778 (44.9)	60,771 (43.7)	84,236 (41.1)	73,909 (40.8)	101,061 (38.5)	83,361 (40.2)	113,439 (38.1)	92,011 (39.8)	125,216 (37.9)	100,428 (39.2)	136,009 (37.3)	108,998 (38.0)	146,677 (36.2)	116,348 (37.0)	156,037 (35.3)	124,280 (37.0)	166,361 (35.2)
East Coast	3,530 (19.7)	5,746 (21.1)	8,674 (22.5)	15,101 (24.4)	22,310 (16.0)	36,298 (17.7)	27,252 (15.0)	44,213 (16.9)	31,410 (15.1)	50,473 (16.9)	35,136 (15.2)	56,275 (17.0)	38,735 (15.1)	62,051 (17.0)	43,304 (15.1)	69,343 (17.1)	47,532 (15.1)	76,027 (17.2)	49,698 (14.8)	79,990 (16.9)
Northern	3,329 (18.6)	5,204 (19.1)	8,332 (21.6)	13,648 (22.0)	35,331 (25.4)	53,589 (26.1)	50,614 (27.9)	74,324 (28.3)	58,490 (28.2)	85,246 (28.6)	64,961 (28.1)	93,741 (28.3)	73,444 (28.7)	105,541 (29.0)	86,933 (30.3)	122,841 (30.3)	98,751 (31.4)	138,678 (31.4)	107,260 (31.9)	150,371 (31.8)
Southern	1,576 (8.8)	2,651 (9.7)	3,316 (8.6)	5,405 (8.7)	20,610 (14.8)	30,959 (15.1)	29,511 (16.3)	42,770 (16.3)	34,180 (16.5)	48,966 (16.4)	39,277 (17.0)	55,542 (16.8)	43,611 (17.0)	60,774 (16.7)	47,713 (16.6)	65,935 (16.3)	51,583 (16.4)	71,207 (16.1)	54,928 (16.3)	75,830 (16.0)
**Ethnicity, n (%)**																				
Bumiputera	10,427 (58.2)	17,683 (64.9)	24,122 (62.4)	43,159 (69.7)	82,706 (59.5)	134,975 (65.8)	107,482 (59.3)	171,738 (65.5)	122,895 (59.2)	195,079 (65.4)	137,710 (59.5)	217,118 (65.6)	154,294 (60.2)	241,525 (66.3)	174,999 (61.0)	270,563 (66.8)	193,742 (61.7)	298,023 (67.4)	208,748 (62.1)	320,313 (67.8)
Chinese	4,398 (24.5)	5,049 (18.5)	8,803 (22.8)	10,096 (16.3)	33,235 (23.9)	37,307 (18.2)	43,550 (24.0)	48,200 (18.4)	50,208 (24.2)	54,823 (18.4)	55,317 (23.9)	59,685 (18.0)	60,205 (23.5)	64,171 (17.6)	66,066 (23.0)	69,698 (17.2)	71,129 (22.6)	74,612 (16.9)	75,071 (22.3)	78,444 (16.6)
Indian	3,095 (17.3)	4,524 (16.6)	5,711 (14.8)	8,677 (14.0)	23,081 (16.6)	32,800 (16.0)	30,254 (16.7)	42,430 (16.2)	34,338 (16.6)	48,222 (16.2)	38,358 (16.6)	53,971 (16.3)	41,719 (16.3)	58,679 (16.1)	45,883 (16.0)	64,535 (15.9)	49,343 (15.7)	69,314 (15.7)	52,347 (15.6)	73,795 (15.6)
Smoking in that particular year, n (%)	1,370 (7.6)	115 (0.4)	3,304 (8.6)	228 (0.4)	16,701 (12.0)	1,026 (0.5)	22,061 (12.2)	1,305 (0.5)	25,128 (12.1)	1,450 (0.5)	28,081 (12.1)	1,594 (0.5)	31,510 (12.3)	1,724 (0.5)	35,297 (12.3)	1,891 (0.5)	38,131 (12.1)	2,006 (0.5)	40,124 (11.9)	2,115 (0.4)
Hyper-tension in that particular year, n (%)	10,204 (56.9)	16,753 (61.5)	23,238 (60.1)	39,970 (64.5)	91,106 (65.5)	145,463 (70.9)	116,670 (64.4)	184,266 (70.2)	131,911 (63.6)	207,692 (69.7)	145,721 (63.0)	228,496 (69.1)	160,237 (62.5)	250,368 (68.7)	179,545 (62.6)	277,309 (68.5)	195,954 (62.4)	301,218 (68.2)	207,622 (61.8)	318,655 (67.4)
Dys-lipidemia in that particular year, n (%)	7,226 (40.3)	12,098 (44.4)	17,050 (44.1)	29,756 (48.0)	67,435 (48.5)	109,299 (53.3)	87,525 (48.3)	139,357 (53.1)	100,226 (48.3)	158,214 (53.1)	112,316 (48.5)	175,750 (53.1)	125,349 (48.9)	194,453 (53.4)	142,940 (49.8)	218,829 (54.1)	158,688 (50.5)	240,674 (54.5)	170,358 (50.7)	257,056 (54.4)
Prior CVD in that particular year, n (%)	1,187 (6.6)	1,214 (4.5)	2,804 (7.3)	3,075 (5.0)	11,432 (8.2)	11,682 (5.7)	14,370 (7.9)	14,255 (5.4)	15,523 (7.5)	15,244 (5.1)	16,503 (7.1)	15,876 (4.8)	17,743 (6.9)	16,586 (4.6)	20,157 (7.0)	17,963 (4.4)	22,109 (7.0)	18,986 (4.3)	22,954 (6.8)	19,291 (4.1)
IHD	937 (5.2)	936 (3.4)	2,249 (5.8)	2,411 (3.9)	8,717 (6.3)	8,933 (4.4)	10,996 (6.1)	10,922 (4.2)	11,926 (5.7)	11,715 (3.9)	12,719 (5.5)	12,192 (3.7)	13,630 (5.3)	12,705 (3.5)	15,628 (5.4)	13,734 (3.4)	17,228 (5.5)	14,527 (3.3)	17,892 (5.3)	14,662 (3.1)
Stroke	223 (1.2)	237 (0.9)	514 (1.3)	572 (0.9)	2,384 (1.7)	2,427 (1.2)	3,019 (1.7)	2,948 (1.1)	3,219 (1.6)	3,111 (1.0)	3,399 (1.5)	3,222 (1.0)	3,702 (1.4)	3,371 (0.9)	4,128 (1.4)	3,691 (0.9)	4,466 (1.4)	3,912 (0.9)	4,645 (1.4)	4,082 (0.9)
Amputation	94 (0.5)	91 (0.3)	185 (0.5)	200 (0.3)	964 (0.7)	834 (0.4)	1,145 (0.6)	997 (0.4)	1,193 (0.6)	1,051 (0.4)	1,242 (0.5)	1,093 (0.3)	1,313 (0.5)	1,135 (0.3)	1,422 (0.5)	1,215 (0.3)	1,517 (0.5)	1,252 (0.3)	1,534 (0.5)	1,259 (0.3)

Footnotes: Data are expressed as mean ± standard deviation, median (interquartile range)^#^ or number (percentage), as appropriate. Cardiovascular disease (CVD) was defined as the presence of either ischemic heart disease, stroke or amputation at registration in the registry. The definitions of hypertension and dyslipidemia were according to local treatment guidelines during the time periods. The Bumiputera ethnicity is a term used in Malaysia to describe Malays and other native people. IHD, ischemic heart disease; n (%), number (percentage).

Fig. 1 and Table S2 summarize the trends in all-cause mortality rates between 2010 and 2019, standardized by age, sex, and duration of type 2 diabetes. The mortality rates among all males with type 2 diabetes did not change over time, showing an AAPC of −0.02 % (95 % CI −0.64 %, 0.61 %). Among males aged 40–49 years and 50–59 years, the mortality rate increased with an AAPC of 2.46 % (95 % CI 0.42 %, 4.55 %) and 1.91 % (95 % CI 0.73 %, 3.10 %), respectively. In all females, all-cause mortality rates were also stable over time, showing an AAPC of −0.49 % (95 % CI −1.57 %, 0.60 %). The mortality rate increased among females aged 40–49 years, although the rates did not change in females aged 50–59 and 60–69 years. In both sexes, there was a significant decrease in mortality rate among those aged 70–79 years: an AAPC of −1.13 % (95 % CI −1.67 %, −0.58 %) in males and −1.70 % (95 % CI −3.01 %, −0.37 %) in females.

**Fig. 2:**
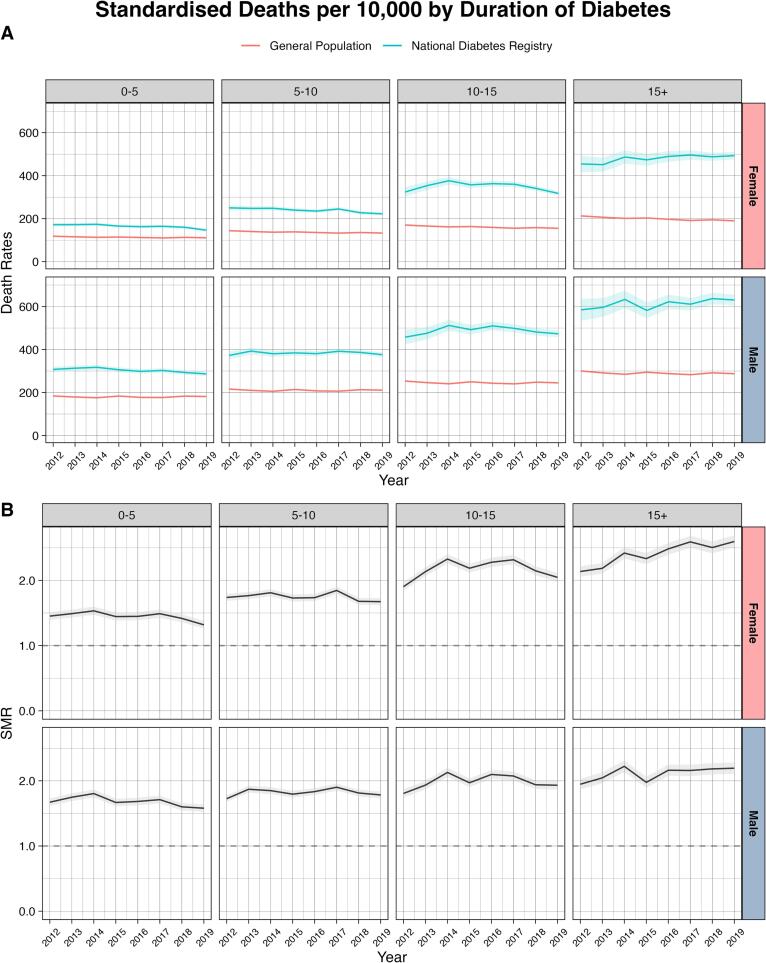
Trends in age-standardized all-cause mortality rates (A) and standardized mortality ratios (B) in people aged 40–79 years with diagnosed type 2 diabetes, stratified by duration of diabetes in that particular year (five-year bands) Footnotes: For mortality rates, confidence intervals were estimated using the Wald normal approximation interval and multiplied by 10,000. For standardized mortality ratio, confidence intervals were estimated using the Breslow and Day method.

Among people who had type 2 diabetes for > 15 years, standardized mortality rates increased over time (Fig. 2 and Table S2). In males with > 15 years duration of type 2 diabetes, the mortality rate significantly increased, showing an AAPC of 1.12 % (95 % CI 0.55 %, 1.69 %). The corresponding AAPC was 1.54 % (95 % CI 0.70 %, 2.38 %) in females. In both sexes, there was a significant decrease in mortality rate among those with < 5 years duration of type 2 diabetes, showing an AAPC of −1.04 % (95 % CI −1.60 %, −0.47 %) in males and −1.82 % (95 % CI −2.82 %, −0.81 %) in females. In addition, females with 5–10 years duration of type 2 diabetes reported a decreasing trend in mortality rate with an AAPC of −1.34 % (95 % CI −2.02 %, −0.65 %).

**Fig. 3:**
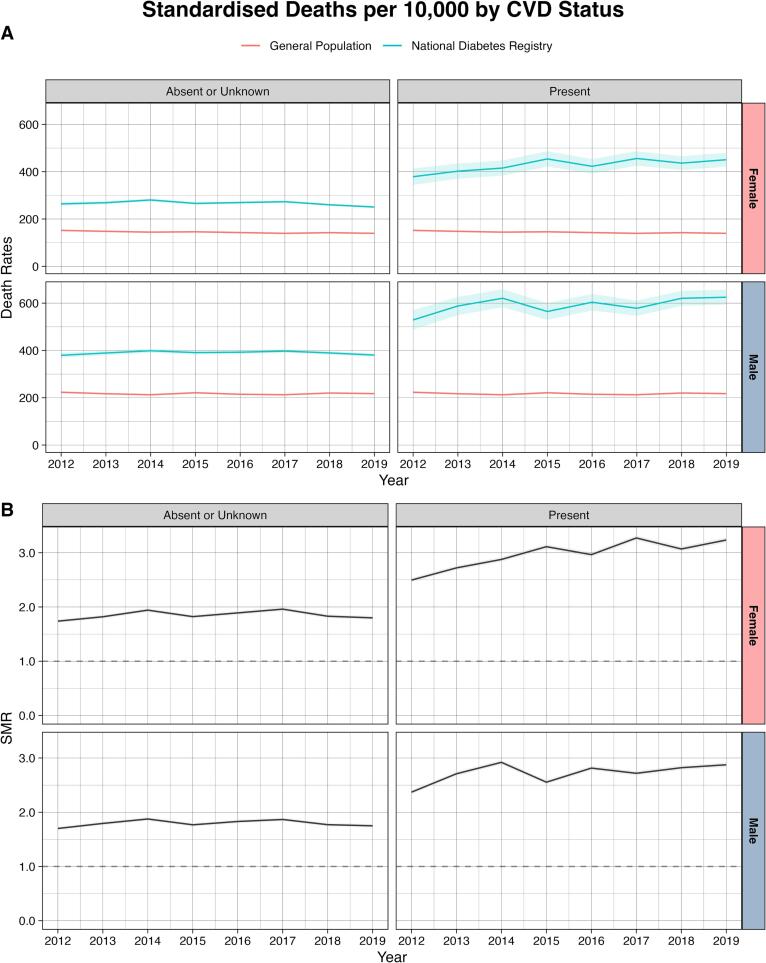
Trends in age-standardized all-cause mortality rates (A) and standardized mortality ratios (B) in people aged 40–79 years with diagnosed type 2 diabetes, stratified by prior history of cardiovascular disease (CVD) Footnotes: For mortality rates, confidence intervals were estimated using the Wald normal approximation interval and multiplied by 10,000. For standardized mortality ratio, confidence intervals were estimated using the Breslow and Day method.

Standardized mortality rates in both sexes with prior CVD increased significantly over time, showing an AAPC of 3.40 % (95 % CI 0.79 %, 6.07 %) in males and 3.01 % (95 % CI 1.65 %, 4.38 %) in females (Fig. 3 and Table S2). Most subgroups stratified by ethnicity or geographical region showed no change in mortality rates, except for females of Indian ethnicity (AAPC −1.83 % [95 % CI −3.37 %, −0.27 %]), males in Southern region (AAPC 1.09 % [95 % CI 0.35 %, 1.84 %]), and females in East Coast region (AAPC −0.97 % [95 % CI −1.76 %, −0.18 %]).

**Fig. 4:**
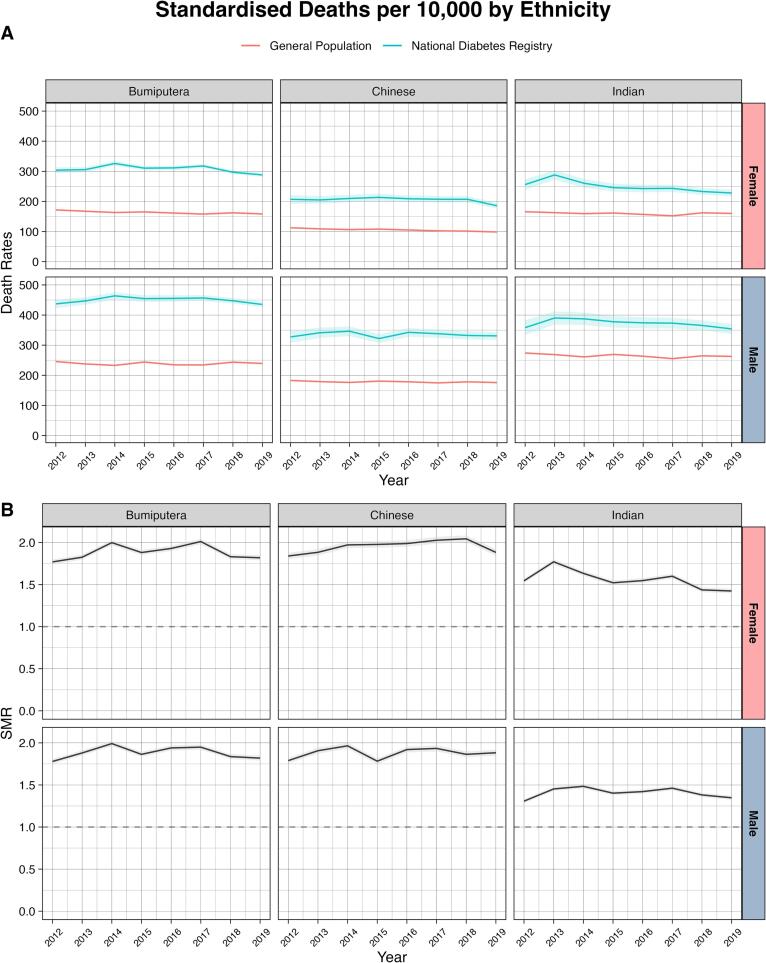
Trends in age-standardized all-cause mortality rates (A) and standardized mortality ratios (B) in people aged 40–79 years with diagnosed type 2 diabetes, stratified by ethnicity Footnotes: For mortality rates, confidence intervals were estimated using the Wald normal approximation interval and multiplied by 10,000. For standardized mortality ratio, confidence intervals were estimated using the Breslow and Day method. The Bumiputera ethnicity is a term used in Malaysia to describe Malays and other native people.

In 2019, compared with the general population, the overall SMR for people with diagnosed type 2 diabetes was 1.83 (95 % CI 1.80, 1.86) for males and 1.85 (95 % CI 1.82, 1.89) for females (Table S3). In both sexes, the SMRs were higher among younger age groups (Fig. 1 and Table S3), those with a longer duration of type 2 diabetes (Fig. 2 and Table S3), and those with prior CVD (Fig. 3 and Table S3). Compared with other ethnicities, SMRs were lower among those of Indian ethnicity in both sexes (Fig. 4 and Table S3). Compared with Bumiputera ethnicity, Chinese and Indian ethnicities had an odds ratio of 0.73 (95 % CI 0.72–0.74) and 0.86 (95 % CI 0.85–0.88), respectively, for mortality, after adjusting for age and sex. The observed patterns in all-cause mortality rates and SMRs were consistent in our sensitivity analyses 1) when we only included primary care clinics that were present in all years from 2012 to 2018 (Table S4 and Table S5), and 2) when we excluded patients who were diagnosed with type 2 diabetes before the age of 30 (Table S6 and Table S7).

## 4 Discussion

The present sample comprised almost one million West Malaysian adults aged 40–79 with diagnosed type 2 diabetes from a primary care-based diabetes registry that collected clinical data that was linked to mortality information for a period of up to 10 years. In contrast to high-income countries, this unique study of a middle-income country reported no evidence that standardized mortality rates for people with type 2 diabetes have decreased over the last 10 years. Indeed, standardized mortality rates increased in several subgroups, including younger people, those with prior CVD, and those who have had type 2 diabetes for > 15 years. This highlights the need for cost-effective interventions to improve diabetes care in Malaysia.

Compared with the Malaysian general population, overall SMR was 1.83 for males and 1.85 for females with type 2 diabetes, and these were stable over the 10-year period. In a retrospective analysis involving 19 data sources from 16 high-income countries/regions in Australia, Asia, Europe, and North America, most of them reported a declining trend in mortality rates with a change in AAPC from −0.5 % in Hungary to −4.2 % in Hong Kong [9]. Besides, 16 data sources reported an overall SMR of < 1.5 and eight of them had a SMR decrease of up to 3 % [9]. On the other hand, the overall rate ratios for all-cause mortality among people with type 2 diabetes in Mexico (also an upper middle-income country) were higher than our findings [33].

Although mortality rates were stable over time in the total population, mortality increased significantly between 2010 and 2019 in people of middle age, those with a longer duration of diabetes, and those with prior CVD: groups that were at substantially elevated risk across the study period. This is in line with a systematic review of 35 observational studies which reported lower rate ratios for all-cause mortality in all age groups with type 2 diabetes, except for younger people aged < 44 years [3]. Of note, high-income countries in East and Southeast Asia reported a declining rate of all-cause mortality than other regions [9], likely driven by decade-long investments in service delivery models, surveillance systems, and increased use of guideline-directed medical therapies including renin-angiotensin system (RAS) inhibitors and statins [34], [35], [36]. Since 2015, the Malaysian government has set priorities for improving care for non-communicable diseases including type 2 diabetes [37]. By leveraging these efforts, further investigations of our findings can be facilitated by enhanced data linkage between NDR and other data sources (if available) in order to understand gaps in care and mortality. These include trends in lifestyle patterns (such as smoking, diet, physical activity level), cardiometabolic risk factors, use of guideline-directed medical therapies (RAS inhibitors, statins, sodium-glucose cotransporter-2 [SGLT2] inhibitors, and glucagon-like peptide-1 receptor agonists [GLP1-RAs]), time lags in implementing evidence-based practice, and the feasibility of integrating structured diabetes assessment programs [10], [38], [39]. Linkage with other Malaysian registries such as the National Cardiovascular Disease Database and hospitalization data would enable a better understanding of the degree to which mortality is due to either high rates of complications or rates of mortality following clinical events.

One of the options for enhancing the treatment of people with type 2 diabetes in Malaysia is to widen access to guideline-directed medical therapies that have been shown to reduce mortality [39]. Although the NDR reported that 82 % of people with type 2 diabetes were treated with statin therapy [40], only simvastatin is currently on the Malaysian Government Essential Medicines list [41]. Switching people with type 2 diabetes at high- and very high cardiorenal risk to generic versions of more potent statins is likely to reduce mortality at modest costs. Similarly, only 53 % of people with type 2 diabetes had angiotensin-converting enzyme inhibitors in NDR 2020 report [40]. To this end, wider use of blood pressure-lowering medications (such as RAS inhibitors) and early combination therapy in those with stage 2 hypertension or beyond (blood pressure ≥ 160/100 mmHg) [42], addressing social determinants of health, and increasing access to newer organ-protective medications such as SGLT2 inhibitors and GLP1-RAs in the public healthcare setting may bring about further reductions in excess mortality risk [10], [37], [43].

Our country-specific analysis of around one million people with type 2 diabetes can provide important insights into the population-level outcomes, given that 74 % of those with diagnosed diabetes received treatment at publicly-funded primary care clinics and two-thirds of them were registered in the present large primary care-based NDR [19]. Together with data linkage to the death registry, this analysis allows a good representation of 10-year trends in mortality estimates at the national public healthcare setting with minimal selection bias. Of note, this is the first analysis reporting mortality rates among people with type 2 diabetes in Malaysia, a middle-income country in Asia, and we also highlight differences by age group, sex, duration of type 2 diabetes, and ethnicity. In terms of ethnic disparities, people with type 2 diabetes of Bumiputera ethnicity had an increased risk of all-cause mortality than Chinese and Indians in Malaysia. This was in line with a study conducted in Singapore with a similar mix of ethnic groups [44]. These findings underpin the need for understanding biological, environmental, psychosocial, and behavioral factors that contribute to the disparity in diabetes care in order to identify effective, pragmatic preventative, and treatment strategies.

Limitations include that, although Malaysia has a well-established civil registration system, there is a lack of reliable information on the cause of death. This is because of up to 34 % of annual deaths are not medically certified as they occur outside a healthcare facility [26]. Of note, MOH is working on verbal autopsy data which will provide future insights into the causes of death. Second, about one-third of people with type 2 diabetes attending public healthcare facilities were not captured in the NDR and Malaysia does not have a linked electronic medical record system at a national level. Hence, our findings may not be generalizable to the entire nationwide type 2 diabetes population especially those managed in private settings. Third, the present findings may also have limited generalizability to East Malaysia. This can be due to variations in: 1) ethnicity structure (more non-Malay Bumiputera in East Malaysia versus more Malay Bumiputera in West Malaysia with different sociodemographic, health literacy level and genetic predisposition), 2) demographic distribution (46 % are living in rural areas in East Malaysia versus the national average of 23 %), and 3) access to health care (accessible to 70 % of population in East Malaysia versus > 95 % in West Malaysia) [18]. Future studies for mortality trends in East Malaysia will provide additional insights when the verbal autopsy data is mature. Fourth, we were not able to link to nationwide hospitalization data. Malaysia is undergoing a new wave of digitalization and the country awaits the fully released version of the Malaysian Health Data Warehouse comprising inpatient and outpatient visits encompassing both public and private healthcare facilities [45]. Fifth, given a lack of robust socioeconomic measures, we were not able to examine the influence of deprivation on trends in mortality among people with type 2 diabetes in Malaysia. Sixth, due to the transition of NDR from paper-based records to a web-based data collection system in 2011, there was an increase in the number of people registered into the NDR. Although the denominator had increased, we did not observe a significant reduction in mortality rates in the main and subgroup analyses, which was in contrast with high-income countries. Lastly, we assumed that all people with no death registration were still alive; if some had died abroad, the true mortality could be marginally higher than our estimates. During our study period, the NDR expanded to include additional clinics (often in rural areas); we minimized the risk of confounding by standardizing by age, sex, and duration of type 2 diabetes, and observed identical trends among clinics that were in the NDR for the entire study period. However, confounding by unobserved variables cannot be ruled out.

## 5 Conclusions

Unlike high-income countries, there has been little to no improvement in mortality for people with type 2 diabetes in Malaysia. Mortality has increased among younger people, those of Bumiputera ethnicity, those with a longer duration of type 2 diabetes, and those with prior CVD. Challenges in the delivery of quality diabetes care need to be identified with concerted follow-up actions from multiple stakeholders in order to reduce the burden of type 2 diabetes and associated healthcare costs in Malaysia.

**Funding:** This work was supported by the UK-Malaysia Joint Partnership on Non-Communicable Diseases under the Malaysia Partnership and Alliances in Research (MyPAiR), funded by the Ministry of Higher Education, Malaysia (Grant number: IF076-2019) and Medical Research Council, United Kingdom. HD and JB are partly funded by the National Institute of Health Research (NIHR) Oxford Biomedical Research Centre. The funders had no role in the study design, data collection and analysis, decision to publish, or preparation of the manuscript. The views and opinions expressed therein are those of the authors and do not necessarily reflect those of the UK NIHR, National Health Service, or the Department of Health. For the purpose of Open Access, the authors have applied a CC BY public copyright licence to any Author Accepted Manuscript version arising from this submission.

## Declaration of Competing Interest

The authors declare the following financial interests/personal relationships which may be considered as potential competing interests: LLL reported receiving research grants and/or honoraria for participating in speaker bureaus from Abbott, AstraZeneca, Boehringer Ingelheim, Merck Sharp & Dohme, Novo Nordisk, Pfizer, Procter & Gamble Health, Roche, Sanofi, Servier, and Zuellig Pharma Therapeutics. Other authors declared no potential conflict of interest.
