# Supplementary material for: Trends in all-cause mortality among adults with diagnosed type 2 diabetes in West Malaysia: 2010 – 2019
Source: Diabetes Res Clin Pract. 2023 Nov;205:110944. doi: 10.1016/j.diabres.2023.110944 (PMC10701628; doi:10.1016/j.diabres.2023.110944)
Supplement: Supplementary data 1 [file mmc1.docx]

**Trends in all-cause mortality among adults with diagnosed type 2 diabetes in West Malaysia: 2010 – 2019**

**Supplementary Information**

**Table of Contents**

[**S1 Text.** Calculations for mortality rates in the National Diabetes Registry population and the general population in Malaysia 2](#_Toc146237136)

[**Figure S1.** Flow chart describing the numerator and denominator included in the all-cause mortality analysis in people with diagnosed type 2 diabetes in Malaysia 3](#_Toc146237137)

[**Table S1.** Proportions of publicly-funded primary care clinics in the Malaysian National Diabetes Registry between 2010 and 2019, stratified by states 4](#_Toc146237138)

[**Table S2.** Trends in age-standardized all-cause mortality rates per 10,000 patient-years among people aged 40-79 years with diagnosed type 2 diabetes in Malaysia 5](#_Toc146237139)

[**Table S3.** Trends in standardized mortality ratios in people aged 40-79 years with diagnosed type 2 diabetes in Malaysia 8](#_Toc146237140)

[**Table S4.** Trends in age-standardized all-cause mortality rates per 10,000 patient-years among people aged 40-79 years with diagnosed type 2 diabetes in Malaysia (for clinics that were included in all years from 2012 to 2018) 10](#_Toc146237141)

[**Table S5.** Trends in standardized mortality ratios in people aged 40-79 years with diagnosed type 2 diabetes in Malaysia (for clinics that were included in all years from 2012 to 2018) 13](#_Toc146237142)

[**Table S6.** Trends in age-standardized all-cause mortality rates per 10,000 patient-years among people aged 40-79 years with diagnosed type 2 diabetes in Malaysia, after excluding those who were diagnosed at younger than 30 years of age 14](#_Toc146237143)

[**Table S7.** Trends in standardized mortality ratios in people aged 40-79 years with diagnosed type 2 diabetes in Malaysia, after excluding those who were diagnosed at younger than 30 years of age 17](#_Toc146237144)

# **S1 Text.** Calculations for mortality rates in the National Diabetes Registry population and the general population in Malaysia

1. **National Diabetes Registry population**

$$Observed proportions of deaths=\frac{number of deaths within subgroup}{total number of people within subgroup}$$

$$Standardised deaths=standardised population \times observed proportions of deaths$$

$$Death rate confidence intervals= Death rate\pm1.96\times\sqrt{\frac{Death rate\times(1-Death rate)}{Frequency}}$$

**b) General population**

$$Annual probability=1-e^{-death rate*}$$

*^*^the death rate was taken from the Malaysia’s abridged life tables for each respective year*

$$Expected number of deaths of general population within subgroup=standardised population within subgroup\times annual probability$$

$$Death rate for general population=\frac{expected number of deaths}{standardised population} \times10,000$$

# **Figure S1.** Flow chart describing the numerator and denominator included in the all-cause mortality analysis in people with diagnosed type 2 diabetes in Malaysia

1,249,147 people with diabetes in registry
(2 Jan 2009-31 Dec 2018)

996,355 people with type 2 diabetes analyzed (Death linkage was censored on

31 Dec 2019)

5,285 (0.42%) excluded due to invalid IC

1,243,862 people with diabetes

and valid IC

247,507 (19.8%) excluded:

- *7,464 (0.6%) Classified as type 1 diabetes*
- *852 (0.07%) Other types of diabetes*
- *673 (0.05%) non-Malaysian*
- *147,805 (11.9%) East Malaysia*
- *29,754 (2.39%) Missing date of diagnosis or mismatch between diagnosis, registration, and death dates*
- *37,434 (3.0%) Aged <40 years*
- *23,525 (1.89%) Aged 80 or above*

Footnotes: IC, identification card number (unique identifier).

# **Table S1.** Proportions of publicly-funded primary care clinics in the Malaysian National Diabetes Registry between 2010 and 2019, stratified by states

| **State** | **2010** | **2011** | **2012** | **2013** | **2014** | **2015** | **2016** | **2017** | **2018** | **2019** |
| --- | --- | --- | --- | --- | --- | --- | --- | --- | --- | --- |
| Johor | 9.3% | 8.6% | 14.9% | 16.2% | 16.4% | 16.8% | 16.7% | 16.4% | 16.2% | 16.1% |
| Kedah | 5.1% | 7.5% | 5.5% | 7.3% | 7.5% | 7.8% | 9.1% | 9.7% | 10.6% | 11.0% |
| Kelantan | 10.3% | 10.6% | 5.2% | 5.1% | 5.1% | 5.1% | 5.1% | 5.0% | 4.9% | 4.7% |
| Melaka | 2.8% | 5.9% | 7.3% | 6.8% | 6.7% | 6.5% | 6.2% | 6.0% | 5.8% | 5.7% |
| Negeri Sembilan | 17.6% | 18.4% | 9.6% | 8.7% | 8.4% | 8.1% | 8.0% | 7.8% | 7.6% | 7.5% |
| Pahang | 7.4% | 8.6% | 7.7% | 7.2% | 7.1% | 7.0% | 6.9% | 6.8% | 6.7% | 6.6% |
| Perak | 7.8% | 8.3% | 11.6% | 12.1% | 12.2% | 11.8% | 11.4% | 12.1% | 12.4% | 12.4% |
| Perlis | 1.2% | 1.1% | 1.7% | 2.2% | 2.2% | 2.1% | 2.0% | 2.0% | 2.0% | 2.0% |
| Pulau Pinang | 4.9% | 5.0% | 7.1% | 6.7% | 6.6% | 6.6% | 6.4% | 6.5% | 6.6% | 6.6% |
| Selangor | 10.2% | 9.9% | 18.6% | 17.6% | 17.8% | 18.2% | 18.3% | 17.6% | 17.2% | 17.1% |
| Terengganu | 2.9% | 4.5% | 4.1% | 3.8% | 4.0% | 4.1% | 4.3% | 4.4% | 4.7% | 4.7% |
| WP Kuala Lumpur | 20.4% | 11.2% | 6.2% | 5.9% | 5.6% | 5.5% | 5.2% | 5.1% | 5.0% | 5.1% |
| WP Putrajaya | 0.2% | 0.4% | 0.4% | 0.4% | 0.4% | 0.4% | 0.4% | 0.5% | 0.5% | 0.6% |
| **Total** | 100.0% | 100.0% | 100.0% | 100.0% | 100.0% | 100.0% | 100.0% | 100.0% | 100.0% | 100.0% |

# **Table S2.** Trends in age-standardized all-cause mortality rates per 10,000 patient-years among people aged 40-79 years with diagnosed type 2 diabetes in Malaysia

|  | | **2010** | **2011** | **2012** | **2013** | **2014** | **2015** | **2016** | **2017** | **2018** | **2019** | **AAPC,**  **%** | **p-value** |
| --- | --- | --- | --- | --- | --- | --- | --- | --- | --- | --- | --- | --- | --- |
| **Overall** | | | | | | | | | | | | | |
| Male | | 412  (383, 441) | 376  (357, 394) | 394  (384, 404) | 408  (399, 417) | 417  (408, 425) | 405  (397, 413) | 409  (402, 417) | 411  (404, 418) | 405  (398, 412) | 397  (390, 404) | -0.02  (-0.64, 0.61) | 0.954 |
| Female | | 260  (241, 278) | 241  (229, 254) | 272  (265, 279) | 278  (271, 284) | 288  (282, 294) | 277  (271, 282) | 278  (272, 283) | 282  (277, 287) | 269  (264, 273) | 258  (254, 263) | -0.49  (-1.57, 0.60) | 0.376 |
| **Age group, years** | | | | | | | | | | | | | |
| Male | 40-49 | 202  (151, 253) | 148  (116, 179) | 155  (139, 171) | 172  (157, 187) | 182  (168, 197) | 192  (177, 206) | 191  (177, 205) | 202  (188, 216) | 202  (189, 216) | 185  (172, 198) | 2.46  (0.42, 4.55) | **0.018*** |
|  | 50-59 | 258  (219, 297) | 221  (196, 246) | 239  (226, 253) | 270  (257, 283) | 279  (267, 292) | 273  (261, 284) | 273  (262, 284) | 286  (275, 296) | 287  (276, 297) | 282  (272, 292) | 1.91  (0.73, 3.10) | **0.002*** |
|  | 60-69 | 429  (378, 480) | 404  (371, 437) | 415  (398, 433) | 427  (411, 442) | 431  (416, 445) | 418  (405, 432) | 417  (404, 429) | 424  (412, 436) | 410  (399, 421) | 410  (400, 421) | -0.40  (-0.82, 0.01) | 0.058 |
|  | 70-79 | 752  (650, 853) | 706  (641, 770) | 744  (709, 778) | 737  (707, 766) | 752  (725, 780) | 718  (692, 743) | 745  (721, 770) | 712  (690, 735) | 709  (688, 729) | 684  (665, 704) | -1.13  (-1.67, -0.58) | **<0.001*** |
| Female | 40-49 | 98  (72, 125) | 60  (46, 75) | 92  (82, 102) | 91  (82, 100) | 104  (95, 113) | 92  (84, 101) | 101  (93, 109) | 106  (98, 115) | 107  (99, 115) | 108  (99, 116) | 3.39  (1.32, 5.50) | **0.001*** |
|  | 50-59 | 158  (134, 182) | 147  (132, 163) | 151  (142, 160) | 167  (159, 175) | 165  (157, 173) | 165  (158, 173) | 163  (156, 170) | 173  (166, 180) | 165  (158, 171) | 158  (152, 164) | 0.43  (-0.80, 1.67) | 0.495 |
|  | 60-69 | 273  (237, 308) | 257  (235, 279) | 277  (265, 290) | 290  (278, 301) | 291  (280, 301) | 281  (271, 290) | 287  (278, 296) | 293  (284, 301) | 279  (271, 287) | 269  (261, 276) | -0.38  (-1.34, 0.60) | 0.448 |
|  | 70-79 | 508  (437, 579) | 488  (442, 533) | 581  (555, 607) | 561  (539, 583) | 608  (587, 629) | 575  (556, 595) | 566  (547, 584) | 557  (540, 574) | 527  (511, 542) | 504  (490, 518) | -1.70  (-3.01, -0.37) | **0.012*** |
| **Duration of diabetes, years** | | | | | | | | | | | | | |
| Male | <5 | 310  (275, 345) | 292  (268, 317) | 308  (295, 320) | 313  (302, 325) | 317  (306, 329) | 306  (295, 317) | 299  (288, 309) | 303  (292, 313) | 293  (283, 303) | 287  (277, 297) | -1.04  (-1.60, -0.47) | **<0.001*** |
|  | 5-10 | 430  (377, 483) | 350  (319, 381) | 373  (355, 390) | 393  (377, 408) | 381  (366, 395) | 384  (371, 398) | 381  (369, 393) | 392  (380, 404) | 387  (375, 398) | 376  (365, 387) | 0.05  (-0.66, 0.75) | 0.898 |
|  | 10-15 | 448  (348, 547) | 444  (381, 507) | 458  (424, 491) | 476  (448, 504) | 512  (487, 538) | 492  (470, 514) | 510  (490, 530) | 498  (480, 517) | 481  (464, 498) | 473  (458, 489) | -0.09  (-1.16, 0.98) | 0.863 |
|  | >15 | 574  (443, 704) | 563  (473, 652) | 585  (534, 636) | 596  (553, 639) | 633  (592, 674) | 582  (546, 618) | 622  (589, 656) | 611  (582, 640) | 637  (610, 664) | 630  (606, 655) | 1.12  (0.55, 1.69) | **<0.001*** |
| Female | <5 | 187  (165, 210) | 161  (146, 175) | 172  (164, 180) | 172  (165, 180) | 174  (166, 181) | 165  (158, 172) | 163  (156, 170) | 165  (158, 171) | 160  (153, 167) | 146  (140, 153) | -1.82  (-2.82, -0.81) | **<0.001*** |
|  | 5-10 | 230  (199, 261) | 233  (214, 253) | 250  (239, 262) | 248  (238, 258) | 248  (239, 258) | 240  (231, 248) | 235  (227, 243) | 245  (237, 253) | 228  (220, 235) | 223  (216, 230) | -1.34  (-2.02, -0.65) | **<0.001*** |
|  | 10-15 | 320  (251, 388) | 277 (237, 316) | 324  (302, 347) | 354  (335, 372) | 377  (359, 394) | 357  (343, 372) | 363  (349, 377) | 360  (348, 373) | 340  (329, 3512) | 317  (307, 328) | -0.94  (-2.85, 1.00) | 0.338 |
|  | >15 | 389  (290, 487) | 377 (313, 441) | 455  (416, 493) | 451  (419, 484) | 487  (456, 517) | 474  (446, 501) | 490  (465, 514) | 496  (474, 517) | 488  (469, 507) | 493  (476, 510) | 1.54  (0.70, 2.38) | **<0.001*** |
| **Prior CVD** | | | | | | | | | | | | | |
| Male | Present | 234  (149, 320) | 379  (309, 450) | 529  (488, 570) | 588  (550, 626) | 621  (583, 659) | 565  (529, 600) | 604  (569, 639) | 578  (546, 610) | 621  (589, 652) | 625  (594, 656) | 3.40  (0.79, 6.07) | **0.011*** |
|  | Absent or Unknown | 429  (398, 459) | 377  (357, 396) | 380  (369, 390) | 389  (380, 398) | 399  (390, 407) | 391  (383, 399) | 393  (385, 400) | 397  (390, 405) | 389  (382, 396) | 380  (374, 387) | -0.16  (-0.77, 0.45) | 0.599 |
| Female | Present | 259  (170, 349) | 314  (253, 376) | 379  (344, 413) | 402  (370, 434) | 415  (384, 447) | 454  (422, 486) | 423  (392, 453) | 456  (426, 486) | 436  (407, 465) | 451  (421, 480) | 3.01  (1.65, 4.38) | **<0.001*** |
|  | Absent or Unknown | 260  (241, 279) | 237  (225, 250) | 264  (257, 271) | 269  (263, 275) | 280  (274, 286) | 266  (260, 271) | 270  (264, 275) | 273  (268, 278) | 260  (255, 265) | 251  (246, 255) | -0.56  (-1.58, 0.48) | 0.290 |
| **Ethnicity** | | | | | | | | | | | | | |
| Male | Bumiputera | 462  (422, 502) | 384  (360, 408) | 437  (423, 451) | 447  (434, 459) | 464  (452, 476) | 455  (444, 466) | 455  (445, 466) | 457  (447, 466) | 447  (438, 457) | 435  (426, 444) | 0.02  (-0.92, 0.96) | 0.971 |
|  | Chinese | 346  (292, 400) | 346  (308, 384) | 327  (308, 346) | 341  (324, 358) | 346  (330, 362) | 322  (307, 337) | 343  (328, 357) | 338  (324, 352) | 332  (319, 345) | 331  (318, 344) | -0.23  (-0.72, 0.26) | 0.350 |
|  | Indian | 396  (328, 465) | 379  (330, 429) | 358  (334, 382) | 390  (368, 412) | 387  (367, 408) | 378  (359, 397) | 374  (356, 392) | 373  (356, 390) | 366  (349, 382) | 354  (338, 370) | -0.88  (-1.81, 0.06) | 0.068 |
|  | Others | 380  (87, 674) | 198  (29, 367) | 293  (170, 416) | 428  (301, 556) | 403  (288, 519) | 436  (323, 549) | 353  (257, 450) | 365  (274, 456) | 456  (359, 552) | 314  (236, 392) | 0.99  (-4.53, 6.83) | 0.731 |
| Female | Bumiputera | 302  (277, 327) | 268  (253, 283) | 304  (295, 313) | 306  (297, 314) | 326  (318, 334) | 310  (303, 318) | 311  (304, 318) | 318  (311, 324) | 297  (291, 303) | 288  (282, 294) | -0.55  (-1.72, 0.63) | 0.361 |
|  | Chinese | 208  (169, 247) | 200  (172, 227) | 207  (193, 221) | 205  (192, 218) | 210  (198, 222) | 213  (202, 225) | 209  (198, 220) | 207  (197, 218) | 207  (197, 217) | 186  (176, 195) | -0.99  (-2.35, 0.39) | 0.158 |
|  | Indian | 247  (201, 292) | 205  (175, 234) | 256  (239, 273) | 288  (272, 304) | 260  (246, 274) | 246  (233, 259) | 243  (230, 255) | 243  (231, 255) | 233  (222, 244) | 228  (217, 239) | -1.83  (-3.37, -0.27) | **0.022*** |
|  | Others | 230  (12, 448) | 130  (12, 247) | 349  (237, 461) | 312  (220, 403) | 268  (188, 347) | 231  (161, 300) | 322  (245, 399) | 268  (202, 335) | 275  (211, 339) | 250  (192, 308) | 0.36  (-4.59, 5.56) | 0.891 |
| **Region** | | | | | | | | | | | | | |
| Male | Central | 382  (344, 421) | 370  (343, 398) | 363  (349, 378) | 384  (370, 398) | 380  (367, 393) | 372  (360, 384) | 381  (369, 393) | 376  (364, 387) | 359  (348, 369) | 358  (348, 368) | -0.65  (-1.31, 0.01) | 0.054 |
|  | East Coast | 523  (450, 596) | 396  (355, 437) | 460  (433, 487) | 492  (467, 518) | 512  (487, 536) | 481  (459, 504) | 496  (474, 517) | 485  (465, 505) | 467  (448, 486) | 452  (434, 471) | -0.36  (-1.87, 1.17) | 0.643 |
|  | Northern | 430  (362, 499) | 357  (317, 397) | 412  (392, 433) | 406  (389, 423) | 423  (407, 440) | 408  (393, 423) | 404  (389, 418) | 416  (402, 429) | 421  (408, 433) | 409  (398, 421) | 0.27  (-0.36, 0.91) | 0.401 |
|  | Southern | 358  (267, 450) | 353  (290, 416) | 384  (358, 410) | 394  (372, 416) | 415  (394, 436) | 408  (388, 427) | 409  (390, 427) | 420  (402, 438) | 423  (406, 441) | 412  (395, 428) | 1.09  (0.35, 1.84) | **0.004*** |
| Female | Central | 234  (209, 259) | 205  (189, 222) | 252  (242, 263) | 260  (250, 270) | 261  (251, 270) | 252  (243, 261) | 250  (241, 258) | 257  (249, 265) | 241  (233, 249) | 225  (218, 232) | -0.87  (-2.51, 0.79) | 0.302 |
|  | East Coast | 357  (309, 404) | 351  (321, 380) | 335  (317, 354) | 322  (305, 338) | 357  (340, 373) | 333  (318, 348) | 335  (321, 349) | 336  (323, 349) | 322  (309, 334) | 315  (303, 327) | -0.97  (-1.76, -0.18) | **0.016*** |
|  | Northern | 284  (239, 329) | 243  (218, 269) | 277  (263, 291) | 268  (257, 280) | 294  (283, 305) | 285  (275, 296) | 290  (280, 300) | 287  (277, 296) | 278  (269, 287) | 266  (257, 274) | -0.25  (-1.46, 0.97) | 0.682 |
|  | Southern | 196  (144, 249) | 221  (182, 260) | 256  (238, 273) | 291  (275, 307) | 279  (264, 293) | 269  (256, 283) | 267  (254, 280) | 275  (263, 288) | 258  (246, 270) | 261  (250, 273) | -0.26  (-1.62, 1.12) | 0.714 |

Footnotes: All numbers in brackets are 95% confidence intervals of either the mortality rates or AAPC. Cardiovascular disease (CVD) was defined as the presence of either ischemic heart disease, stroke, or amputation at registration in the registry. The Bumiputera ethnicity is a term used in Malaysia to describe Malays and other native people.

# **Table S3.** Trends in standardized mortality ratios in people aged 40-79 years with diagnosed type 2 diabetes in Malaysia

|  | | **2010** | **2011** | **2012** | **2013** | **2014** | **2015** | **2016** | **2017** | **2018** | **2019** |
| --- | --- | --- | --- | --- | --- | --- | --- | --- | --- | --- | --- |
| **Overall** | | | | | | | | | | | |
| Male | | 1.71  (1.68, 1.73) | 1.66  (1.63, 1.69) | 1.76  (1.73, 1.79) | 1.88  (1.85, 1.91) | 1.96  (1.93, 1.99) | 1.83  (1.8, 1.86) | 1.91  (1.88, 1.94) | 1.93  (1.9, 1.96) | 1.84  (1.81, 1.87) | 1.83  (1.80, 1.86) |
| Female | | 1.64  (1.61, 1.66) | 1.56  (1.53, 1.59) | 1.79  (1.76, 1.82) | 1.88  (1.85, 1.91) | 2.00  (1.96, 2.03) | 1.90  (1.86, 1.93) | 1.95  (1.92, 1.98) | 2.02  (1.99, 2.06) | 1.89  (1.86, 1.92) | 1.85  (1.82, 1.89) |
| **Age group, years** | | | | | | | | | | | |
| Male | 40-49 | 4.63  (4.33, 4.94) | 3.41  (3.15, 3.68) | 3.58  (3.32, 3.86) | 3.97  (3.69, 4.27) | 4.11  (3.83, 4.41) | 4.10  (3.82, 4.39) | 4.32  (4.03, 4.62) | 4.44  (4.15, 4.74) | 4.26  (3.98, 4.55) | 3.89  (3.62, 4.16) |
|  | 50-59 | 2.60  (2.50, 2.70) | 2.15  (2.06, 2.24) | 2.33  (2.24, 2.42) | 2.68  (2.58, 2.78) | 2.81  (2.70, 2.91) | 2.57  (2.47, 2.66) | 2.72  (2.62, 2.82) | 2.77  (2.67, 2.87) | 2.66  (2.56, 2.76) | 2.60  (2.51, 2.70) |
|  | 60-69 | 1.81  (1.76, 1.86) | 1.75  (1.71, 1.80) | 1.84  (1.79, 1.89) | 1.97  (1.91, 2.02) | 2.04  (1.99, 2.10) | 1.86  (1.81, 1.91) | 1.94  (1.88, 1.99) | 1.95  (1.90, 2.01) | 1.81  (1.76, 1.86) | 1.82  (1.77, 1.87) |
|  | 70-79 | 1.26  (1.23, 1.30) | 1.35  (1.31, 1.39) | 1.43  (1.39, 1.47) | 1.45  (1.41, 1.49) | 1.51  (1.47, 1.55) | 1.43  (1.39, 1.47) | 1.50  (1.45, 1.54) | 1.48  (1.44, 1.53) | 1.44  (1.40, 1.49) | 1.43  (1.39, 1.47) |
| Female | 40-49 | 4.46  (4.11, 4.83) | 2.65  (2.39, 2.93) | 4.08  (3.75, 4.44) | 3.96  (3.64, 4.31) | 4.46  (4.12, 4.82) | 3.90  (3.58, 4.23) | 4.26  (3.93, 4.61) | 4.56  (4.22, 4.93) | 4.42  (4.09, 4.77) | 4.41  (4.08, 4.76) |
|  | 50-59 | 2.79  (2.68, 2.91) | 2.45  (2.35, 2.55) | 2.53  (2.43, 2.64) | 2.84  (2.73, 2.95) | 2.86  (2.74, 2.97) | 2.9  (2.79, 3.02) | 2.74  (2.63, 2.85) | 2.85  (2.74, 2.96) | 2.64  (2.53, 2.75) | 2.54  (2.43, 2.64) |
|  | 60-69 | 1.85  (1.79, 1.90) | 1.78  (1.73, 1.84) | 1.97  (1.91, 2.03) | 2.12  (2.07, 2.18) | 2.19  (2.13, 2.25) | 2.02  (1.96, 2.07) | 2.12  (2.06, 2.18) | 2.15  (2.09, 2.21) | 1.99  (1.94, 2.05) | 1.93  (1.88, 1.99) |
|  | 70-79 | 1.16  (1.12, 1.19) | 1.16  (1.13, 1.20) | 1.41  (1.38, 1.45) | 1.40  (1.37, 1.44) | 1.56  (1.52, 1.60) | 1.49  (1.45, 1.53) | 1.52  (1.48, 1.56) | 1.58  (1.54, 1.63) | 1.48  (1.44, 1.52) | 1.47  (1.43, 1.51) |
| **Duration of diabetes, years** | | | | | | | | | | | |
| Male | <5 | 1.58  (1.52, 1.63) | 1.57  (1.52, 1.63) | 1.67  (1.62, 1.73) | 1.75  (1.69, 1.81) | 1.80  (1.74, 1.87) | 1.67  (1.61, 1.73) | 1.68  (1.63, 1.74) | 1.71  (1.65, 1.77) | 1.60  (1.55, 1.66) | 1.58  (1.52, 1.64) |
|  | 5-10 | 1.84  (1.79, 1.90) | 1.60  (1.55, 1.65) | 1.73  (1.68, 1.78) | 1.87  (1.82, 1.92) | 1.85  (1.80, 1.90) | 1.80  (1.74, 1.85) | 1.83  (1.78, 1.89) | 1.90  (1.85, 1.96) | 1.81  (1.76, 1.87) | 1.78  (1.73, 1.84) |
|  | 10-15 | 1.62  (1.57, 1.68) | 1.73  (1.67, 1.79) | 1.81  (1.74, 1.87) | 1.93  (1.87, 2.00) | 2.13  (2.06, 2.20) | 1.97  (1.90, 2.04) | 2.10  (2.03, 2.17) | 2.07  (2.01, 2.14) | 1.94  (1.87, 2.01) | 1.93  (1.87, 2.00) |
|  | >15 | 1.73  (1.66, 1.81) | 1.85  (1.77, 1.93) | 1.95  (1.87, 2.03) | 2.05  (1.96, 2.13) | 2.22  (2.14, 2.31) | 1.98  (1.90, 2.06) | 2.16  (2.08, 2.25) | 2.16  (2.07, 2.25) | 2.18  (2.10, 2.27) | 2.19  (2.11, 2.28) |
| Female | <5 | 1.53  (1.47, 1.59) | 1.33  (1.28, 1.39) | 1.45  (1.39, 1.51) | 1.49  (1.43, 1.55) | 1.53  (1.47, 1.60) | 1.45  (1.39, 1.51) | 1.45  (1.39, 1.51) | 1.49  (1.43, 1.55) | 1.42  (1.36, 1.48) | 1.32  (1.26, 1.38) |
|  | 5-10 | 1.53  (1.48, 1.58) | 1.59  (1.54, 1.64) | 1.74  (1.69, 1.79) | 1.77  (1.71, 1.82) | 1.81  (1.76, 1.87) | 1.73  (1.68, 1.78) | 1.74  (1.68, 1.79) | 1.85  (1.79, 1.9) | 1.68  (1.63, 1.73) | 1.67  (1.62, 1.73) |
|  | 10-15 | 1.79  (1.73, 1.85) | 1.59  (1.54, 1.65) | 1.90  (1.84, 1.97) | 2.13  (2.07, 2.20) | 2.33  (2.26, 2.40) | 2.19  (2.12, 2.26) | 2.28  (2.21, 2.35) | 2.32  (2.25, 2.39) | 2.15  (2.08, 2.22) | 2.05  (1.98, 2.12) |
|  | >15 | 1.73  (1.66, 1.80) | 1.73  (1.66, 1.80) | 2.14  (2.06, 2.22) | 2.19  (2.10, 2.27) | 2.42  (2.33, 2.51) | 2.33  (2.25, 2.42) | 2.48  (2.40, 2.57) | 2.59  (2.50, 2.68) | 2.50  (2.42, 2.60) | 2.60  (2.51, 2.69) |
| **Prior CVD** | | | | | | | | | | | |
| Male | Present | 0.97  (0.95, 0.99) | 1.68  (1.65, 1.71) | 2.37  (2.34, 2.41) | 2.71  (2.67, 2.75) | 2.92  (2.88, 2.96) | 2.55  (2.52, 2.59) | 2.81  (2.78, 2.85) | 2.72  (2.68, 2.76) | 2.82  (2.78, 2.86) | 2.87  (2.84, 2.91) |
|  | Absent or Unknown | 1.77  (1.75, 1.80) | 1.67  (1.64, 1.7) | 1.70  (1.67, 1.73) | 1.79  (1.76, 1.82) | 1.88  (1.84, 1.91) | 1.77  (1.74, 1.80) | 1.83  (1.80, 1.86) | 1.87  (1.83, 1.90) | 1.77  (1.74, 1.8) | 1.75  (1.72, 1.78) |
| Female | Present | 1.63  (1.61, 1.66) | 2.03  (2.00, 2.06) | 2.50  (2.46, 2.53) | 2.72  (2.68, 2.76) | 2.88  (2.84, 2.92) | 3.11  (3.07, 3.15) | 2.96  (2.92, 3.01) | 3.27  (3.23, 3.32) | 3.07  (3.03, 3.11) | 3.23  (3.19, 3.28) |
|  | Absent or Unknown | 1.64  (1.61, 1.67) | 1.53  (1.50, 1.56) | 1.74  (1.71, 1.77) | 1.82  (1.79, 1.85) | 1.94  (1.91, 1.97) | 1.82  (1.79, 1.85) | 1.89  (1.86, 1.92) | 1.96  (1.93, 1.99) | 1.83  (1.80, 1.86) | 1.8  (1.77, 1.83) |
| **Ethnicity** | | | | | | | | | | | |
| Male | Bumiputera | 1.69  (1.66, 1.71) | 1.55  (1.52, 1.57) | 1.78  (1.75, 1.81) | 1.88  (1.85, 1.91) | 1.99  (1.96, 2.02) | 1.86  (1.83, 1.89) | 1.94  (1.91, 1.97) | 1.95  (1.92, 1.98) | 1.84  (1.81, 1.87) | 1.82  (1.79, 1.85) |
|  | Chinese | 1.75  (1.72, 1.78) | 1.86  (1.82, 1.89) | 1.79  (1.76, 1.82) | 1.91  (1.87, 1.94) | 1.96  (1.93, 2.00) | 1.78  (1.75, 1.82) | 1.92  (1.89, 1.96) | 1.93  (1.90, 1.97) | 1.86  (1.83, 1.9) | 1.88  (1.85, 1.92) |
|  | Indian | 1.41  (1.39, 1.44) | 1.38  (1.35, 1.4) | 1.31  (1.28, 1.33) | 1.45  (1.43, 1.48) | 1.48  (1.46, 1.51) | 1.40  (1.38, 1.43) | 1.42  (1.40, 1.45) | 1.46  (1.44, 1.49) | 1.38  (1.36, 1.41) | 1.35  (1.32, 1.37) |
|  | Others | 2.29  (2.25, 2.33) | 0.99  (0.97, 1.02) | 1.48  (1.45, 1.51) | 2.17  (2.14, 2.21) | 2.11  (2.07, 2.15) | 2.21  (2.18, 2.25) | 1.84  (1.81, 1.87) | 1.95  (1.92, 1.99) | 2.39  (2.36, 2.43) | 1.55  (1.52, 1.58) |
| Female | Bumiputera | 1.58  (1.56, 1.61) | 1.54  (1.51, 1.56) | 1.77  (1.74, 1.80) | 1.83  (1.80, 1.85) | 2.00  (1.97, 2.03) | 1.88  (1.85, 1.91) | 1.93  (1.90, 1.96) | 2.01  (1.98, 2.04) | 1.83  (1.80, 1.86) | 1.82  (1.79, 1.85) |
|  | Chinese | 1.88  (1.84, 1.92) | 1.71  (1.67, 1.74) | 1.84  (1.80, 1.88) | 1.88  (1.85, 1.92) | 1.97  (1.93, 2.01) | 1.98  (1.94, 2.02) | 1.99  (1.95, 2.03) | 2.03  (1.99, 2.07) | 2.04  (2.00, 2.08) | 1.88  (1.84, 1.92) |
|  | Indian | 1.51  (1.48, 1.54) | 1.23  (1.21, 1.26) | 1.54  (1.52, 1.57) | 1.77  (1.74, 1.80) | 1.63  (1.60, 1.66) | 1.52  (1.49, 1.55) | 1.55  (1.52, 1.58) | 1.60  (1.57, 1.63) | 1.44  (1.41, 1.46) | 1.42  (1.40, 1.45) |
|  | Others | 1.36  (1.33, 1.38) | 0.71  (0.69, 0.73) | 1.97  (1.94, 2.00) | 1.75  (1.72, 1.78) | 1.54  (1.51, 1.56) | 1.35  (1.33, 1.38) | 1.94  (1.90, 1.97) | 1.72  (1.69, 1.75) | 1.71  (1.68, 1.74) | 1.46  (1.43, 1.49) |

Footnotes: All numbers in brackets are 95% confidence intervals of standardized mortality ratios. Cardiovascular disease (CVD) was defined as the presence of either ischemic heart disease, stroke, or amputation at registration in the registry. The Bumiputera ethnicity is a term used in Malaysia to describe Malays and other native people.

# **Table S4.** Trends in age-standardized all-cause mortality rates per 10,000 patient-years among people aged 40-79 years with diagnosed type 2 diabetes in Malaysia (for clinics that were included in all years from 2012 to 2018)

|  | | **2012** | **2015** | **2017** | **2019** | **AAPC, %** | **p-value** |
| --- | --- | --- | --- | --- | --- | --- | --- |
| **Overall** | | | | | | | |
| Male | | 390  (379, 400) | 400  (391, 408) | 405  (398, 413) | 388  (382, 395) | -0.14  (-0.78, 0.51) | 0.676 |
| Female | | 269  (262, 276) | 275  (269, 281) | 278  (273, 283) | 253  (248, 257) | -0.62  (-1.81, 0.59) | 0.313 |
| **Age group, years** | | | | | | | |
| Male | 40-49 | 153  (136, 170) | 190  (175, 205) | 202  (188, 217) | 177  (164, 191) | 2.13  (-0.14, 4.45) | 0.066 |
|  | 50-59 | 232  (218, 246) | 270  (258, 282) | 279  (268, 290) | 276  (265, 287) | 2.06  (0.78, 3.36) | **0.002*** |
|  | 60-69 | 415  (396, 434) | 409  (395, 423) | 418  (405, 430) | 402  (390, 413) | -0.56  (-1.04, -0.08) | **0.023*** |
|  | 70-79 | 733  (697, 769) | 713  (687, 739) | 703  (680, 726) | 670  (650, 690) | -1.30  (-1.89, -0.71) | **<0.001*** |
| Female | 40-49 | 88  (78, 99) | 91  (82, 99) | 103  (94, 112) | 105  (96, 114) | 3.70  (1.72, 5.72) | **<0.001*** |
|  | 50-59 | 150  (141, 159) | 160  (152, 168) | 167  (160, 174) | 153  (146, 160) | 0.07  (-1.15, 1.29) | 0.916 |
|  | 60-69 | 271  (258, 284) | 280  (270, 290) | 290  (280, 299) | 262  (254, 270) | -0.39  (-1.54, 0.77) | 0.506 |
|  | 70-79 | 576  (549, 603) | 571  (552, 591) | 549  (532, 567) | 492  (477, 507) | -1.89  (-3.27, -0.50) | **0.008*** |
| **Duration of diabetes, years** | | | | | | | |
| Male | <5 | 305  (292, 318) | 303  (292, 314) | 294  (283, 305) | 276  (265, 286) | -1.39  (-2.08, -0.70) | **<0.001*** |
|  | 5-10 | 369  (351, 387) | 380  (366, 394) | 388  (375, 400) | 366  (354, 377) | -0.12  (-0.83, 0.60) | 0.739 |
|  | 10-15 | 454  (419, 489) | 487  (465, 510) | 493  (474, 512) | 466  (449, 482) | -0.13  (-1.13, 0.87) | 0.797 |
|  | >15 | 567  (516, 618) | 563  (526, 599) | 602  (572, 632) | 625  (600, 651) | 1.32  (0.65, 1.99) | **<0.001*** |
| Female | <5 | 173  (165, 182) | 163  (156, 171) | 161  (154, 168) | 141  (135, 148) | -2.28  (-3.22, -1.33) | **<0.001*** |
|  | 5-10 | 244  (232, 255) | 237  (228, 246) | 242  (234, 251) | 216  (208, 223) | -1.44  (-2.38, -0.5) | **0.003*** |
|  | 10-15 | 320  (297, 343) | 351  (336, 366) | 352  (339, 365) | 309  (298, 320) | -1.03  (-3.01, 0.99) | 0.314 |
|  | >15 | 448  (409, 488) | 475  (447, 504) | 488  (466, 510) | 486  (468, 504) | 1.42  (0.53, 2.33) | **0.002*** |
| **Prior CVD** | | | | | | | |
| Male | Present | 526  (483, 568) | 560  (524, 596) | 561  (527, 596) | 607  (572, 641) | 3.04  (0.41, 5.75) | **0.023*** |
|  | Absent or Unknown | 375  (364, 386) | 386  (378, 395) | 393  (386, 401) | 374  (367, 381) | -0.18  (-0.8, 0.45) | 0.577 |
| Female | Present | 392  (355, 429) | 452  (418, 485) | 451  (418, 484) | 436  (404, 468) | 2.30  (0.59, 4.03) | **0.008*** |
|  | Absent or Unknown | 260  (252, 267) | 264  (258, 270) | 270  (264, 275) | 246  (241, 251) | -0.58  (-1.73, 0.58) | 0.328 |
| **Ethnicity** | | | | | | | |
| Male | Bumiputera | 431  (417, 446) | 448  (437, 460) | 449  (439, 460) | 424  (415, 433) | -0.13  (-1.11, 0.86) | 0.800 |
|  | Chinese | 329  (309, 349) | 322  (307, 337) | 336  (322, 350) | 329  (315, 342) | -0.29  (-0.71, 0.15) | 0.194 |
|  | Indian | 354  (329, 378) | 376  (357, 395) | 375  (357, 393) | 349  (332, 365) | -0.89  (-1.96, 0.19) | 0.107 |
|  | Others | 315  (183, 447) | 420  (305, 534) | 351  (257, 446) | 301  (218, 383) | 1.10  (-4.61, 7.15) | 0.712 |
| Female | Bumiputera | 301  (291, 310) | 309  (301, 317) | 314  (307, 321) | 282  (276, 288) | -0.65  (-1.93, 0.65) | 0.326 |
|  | Chinese | 206  (191, 221) | 214  (202, 226) | 205  (194, 216) | 185  (175, 195) | -1.10  (-2.35, 0.16) | 0.087 |
|  | Indian | 257  (239, 274) | 245  (232, 258) | 244  (232, 256) | 225  (214, 236) | -1.91  (-3.62, -0.18) | **0.031*** |
|  | Others | 331  (216, 446) | 213  (143, 283) | 278  (206, 349) | 241  (179, 303) | 1.79  (-4.33, 8.30) | 0.576 |
| **Region** | | | | | | | |
| Male | Central | 364  (349, 379) | 372  (360, 385) | 377  (366, 389) | 356  (345, 366) | -0.60  (-1.28, 0.10) | 0.091 |
|  | East Coast | 447  (418, 477) | 475  (451, 499) | 476  (454, 498) | 443  (423, 464) | -0.41  (-1.95, 1.16) | 0.611 |
|  | Northern | 412  (390, 434) | 399  (384, 415) | 405  (391, 419) | 398  (386, 411) | -0.09  (-0.72, 0.54) | 0.774 |
|  | Southern | 373  (346, 401) | 405  (385, 425) | 418  (400, 437) | 402  (385, 420) | 1.09  (0.15, 2.04) | **0.023*** |
| Female | Central | 253  (242, 263) | 253  (244, 261) | 257  (248, 265) | 225  (218, 232) | -0.83  (-2.5, 0.86) | 0.335 |
|  | East Coast | 335  (315, 355) | 332  (316, 347) | 332  (317, 347) | 314  (301, 327) | -1.07  (-2.00, -0.13) | **0.025*** |
|  | Northern | 271  (257, 286) | 283  (272, 294) | 282  (272, 292) | 256  (247, 265) | -0.56  (-1.94, 0.84) | 0.433 |
|  | Southern | 254  (236, 273) | 267  (254, 281) | 273  (260, 286) | 255  (243, 266) | -0.38  (-1.64, 0.91) | 0.566 |

Footnotes: All numbers in brackets are 95% confidence intervals of either the mortality rates or AAPC. Cardiovascular disease (CVD) was defined as the presence of either ischemic heart disease, stroke, or amputation at registration into the registry. The Bumiputera ethnicity is a term used in Malaysia to describe Malays and other native people. AAPC, average annual percent change.

# **Table S5.** Trends in standardized mortality ratios in people aged 40-79 years with diagnosed type 2 diabetes in Malaysia (for clinics that were included in all years from 2012 to 2018)

|  | | **2012** | **2015** | **2017** | **2019** |
| --- | --- | --- | --- | --- | --- |
| **Overall** | | | | | |
| Male | | 1.74 (1.71, 1.77) | 1.80 (1.77, 1.84) | 1.90 (1.87, 1.93) | 1.78 (1.75, 1.82) |
| Female | | 1.76 (1.73, 1.79) | 1.87 (1.84, 1.91) | 1.98 (1.95, 2.02) | 1.80 (1.77, 1.84) |
| **Age group, years** | | | | | |
| Male | 40-49 | 3.54 (3.26, 3.84) | 4.06 (3.77, 4.37) | 4.44 (4.13, 4.76) | 3.72 (3.45, 4.01) |
|  | 50-59 | 2.26 (2.16, 2.36) | 2.55 (2.44, 2.65) | 2.71 (2.6, 2.81) | 2.54 (2.44, 2.65) |
|  | 60-69 | 1.84 (1.79, 1.90) | 1.82 (1.76, 1.87) | 1.92 (1.87, 1.98) | 1.78 (1.73, 1.84) |
|  | 70-79 | 1.41 (1.37, 1.45) | 1.42 (1.38, 1.47) | 1.46 (1.42, 1.51) | 1.40 (1.36, 1.44) |
| Female | 40-49 | 3.92 (3.58, 4.29) | 3.83 (3.50, 4.19) | 4.43 (4.07, 4.81) | 4.30 (3.95, 4.67) |
|  | 50-59 | 2.52 (2.41, 2.64) | 2.81 (2.69, 2.93) | 2.76 (2.64, 2.88) | 2.46 (2.35, 2.57) |
|  | 60-69 | 1.92 (1.86, 1.98) | 2.01 (1.95, 2.07) | 2.12 (2.06, 2.19) | 1.88 (1.83, 1.94) |
|  | 70-79 | 1.40 (1.36, 1.44) | 1.48 (1.43, 1.52) | 1.56 (1.51, 1.61) | 1.43 (1.39, 1.48) |
| **Duration of diabetes, years** | | | | | |
| Male | <5 | 1.66 (1.60, 1.72) | 1.65 (1.59, 1.72) | 1.66 (1.60, 1.72) | 1.52 (1.46, 1.58) |
|  | 5-10 | 1.71 (1.66, 1.76) | 1.78 (1.72, 1.83) | 1.88 (1.83, 1.94) | 1.74 (1.68, 1.79) |
|  | 10-15 | 1.79 (1.72, 1.85) | 1.94 (1.88, 2.01) | 2.05 (1.97, 2.12) | 1.90 (1.83, 1.97) |
|  | >15 | 1.88 (1.80, 1.97) | 1.90 (1.82, 1.99) | 2.12 (2.03, 2.21) | 2.17 (2.08, 2.26) |
| Female | <5 | 1.46 (1.40, 1.53) | 1.43 (1.36, 1.49) | 1.46 (1.39, 1.52) | 1.27 (1.21, 1.34) |
|  | 5-10 | 1.69 (1.63, 1.74) | 1.71 (1.65, 1.76) | 1.82 (1.76, 1.88) | 1.62 (1.56, 1.68) |
|  | 10-15 | 1.87 (1.81, 1.94) | 2.14 (2.07, 2.21) | 2.26 (2.18, 2.33) | 1.99 (1.92, 2.06) |
|  | >15 | 2.09 (2.01, 2.17) | 2.32 (2.23, 2.41) | 2.53 (2.43, 2.63) | 2.54 (2.44, 2.64) |
| **Prior CVD** | | | | | |
| Male | Present | 2.35 (2.31, 2.39) | 2.53 (2.49, 2.57) | 2.63 (2.59, 2.67) | 2.78 (2.74, 2.83) |
|  | Absent or Unknown | 1.68 (1.65, 1.71) | 1.74 (1.71, 1.78) | 1.84 (1.81, 1.88) | 1.72 (1.69, 1.75) |
| Female | Present | 2.57 (2.53, 2.61) | 3.08 (3.03, 3.12) | 3.22 (3.17, 3.27) | 3.11 (3.07, 3.16) |
|  | Absent or Unknown | 1.70 (1.67, 1.73) | 1.80 (1.77, 1.83) | 1.93 (1.89, 1.96) | 1.76 (1.72, 1.79) |
| **Ethnicity** | | | | | |
| Male | Bumiputera | 1.75 (1.72, 1.78) | 1.83 (1.80, 1.86) | 1.91 (1.88, 1.95) | 1.77 (1.74, 1.80) |
|  | Chinese | 1.79 (1.76, 1.83) | 1.78 (1.75, 1.82) | 1.92 (1.88, 1.96) | 1.87 (1.83, 1.90) |
|  | Indian | 1.29 (1.27, 1.31) | 1.39 (1.37, 1.42) | 1.47 (1.44, 1.50) | 1.33 (1.30, 1.35) |
|  | Others | 1.59 (1.56, 1.62) | 2.12 (2.09, 2.16) | 1.87 (1.84, 1.91) | 1.48 (1.45, 1.51) |
| Female | Bumiputera | 1.74 (1.71, 1.77) | 1.86 (1.83, 1.89) | 1.98 (1.95, 2.01) | 1.77 (1.74, 1.81) |
|  | Chinese | 1.82 (1.78, 1.86) | 1.97 (1.93, 2.01) | 1.99 (1.95, 2.04) | 1.87 (1.82, 1.91) |
|  | Indian | 1.54 (1.51, 1.57) | 1.51 (1.48, 1.54) | 1.60 (1.57, 1.63) | 1.40 (1.37, 1.43) |
|  | Others | 1.86 (1.83, 1.89) | 1.24 (1.22, 1.27) | 1.77 (1.73, 1.80) | 1.40 (1.37, 1.43) |

Footnotes: All numbers in brackets are 95% confidence intervals of standardized mortality ratios. Cardiovascular disease (CVD) was defined as the presence of either ischemic heart disease, stroke, or amputation at registration into the registry. The Bumiputera ethnicity is a term used in Malaysia to describe Malays and other native people.

# **Table S6.** Trends in age-standardized all-cause mortality rates per 10,000 patient-years among people aged 40-79 years with diagnosed type 2 diabetes in Malaysia, after excluding those who were diagnosed at younger than 30 years of age

|  | | **2012** | **2015** | **2017** | **2019** | **AAPC, %** | **p-value** |
| --- | --- | --- | --- | --- | --- | --- | --- |
| **Overall** | | | | | | | |
| Male | | 393  (383, 404) | 404  (396, 412) | 410  (403, 417) | 397  (390, 403) | -0.04  (-0.65, 0.57) | 0.900 |
| Female | | 272  (265, 279) | 276  (270, 281) | 282  (277, 287) | 258  (253, 262) | -0.49  (-1.57, 0.59) | 0.372 |
| **Age group, years** | | | | | | | |
| Male | 40-49 | 146  (130, 162) | 184  (170, 199) | 195  (181, 209) | 179  (167, 192) | 2.71  (0.50, 4.98) | **0.016*** |
|  | 50-59 | 238  (225, 252) | 270  (259, 282) | 284  (273, 295) | 281  (270, 291) | 1.88  (0.74, 3.03) | **0.001*** |
|  | 60-69 | 415  (397, 433) | 417  (404, 431) | 423  (411, 435) | 410  (399, 421) | -0.43  (-0.83, -0.03) | **0.034*** |
|  | 70-79 | 743  (708, 778) | 718  (692, 743) | 712  (690, 734) | 684  (665, 703) | -1.14  (-1.68, -0.59) | **<0.001*** |
| Female | 40-49 | 87  (78, 97) | 86  (77, 94) | 102  (93, 110) | 102  (94, 110) | 3.41  (1.70, 5.15) | **<0.001*** |
|  | 50-59 | 151  (142, 159) | 163  (155, 170) | 172  (165, 179) | 157  (150, 163) | 0.45  (-0.72, 1.63) | 0.455 |
|  | 60-69 | 275  (263, 288) | 280  (270, 290) | 292  (284, 301) | 268  (261, 276) | -0.37  (-1.37, 0.64) | 0.470 |
|  | 70-79 | 582  (556, 608) | 576  (556, 595) | 557  (540, 574) | 504  (490, 518) | -1.69  (-3.00, -0.37) | **0.013*** |
| **Duration of diabetes, years** | | | | | | | |
| Male | <5 | 308  (295, 320) | 306  (295, 317) | 303  (292, 313) | 287  (277, 297) | -1.04  (-1.60, -0.47) | **<0.001*** |
|  | 5-10 | 373  (355, 390) | 384  (371, 398) | 392  (380, 404) | 376  (365, 387) | 0.05  (-0.66, 0.75) | 0.900 |
|  | 10-15 | 458  (424, 492) | 493  (471, 515) | 500  (481, 518) | 475  (459, 491) | -0.11  (-1.17, 0.96) | 0.840 |
|  | >15 | 588  (535, 641) | 580  (542, 617) | 609  (580, 639) | 635  (610, 660) | 1.10  (0.54, 1.66) | **<0.001*** |
| Female | <5 | 172  (164, 180) | 165  (158, 172) | 165  (158, 171) | 146  (140, 153) | -1.83  (-2.83, -0.82) | **<0.001*** |
|  | 5-10 | 250  (239, 262) | 240  (231, 248) | 245  (237, 253) | 223  (216, 230) | -1.34  (-2.02, -0.65) | **<0.001*** |
|  | 10-15 | 326  (304, 349) | 359  (345, 374) | 362  (349, 374) | 318  (308, 328) | -0.99  (-2.90, 0.96) | 0.319 |
|  | >15 | 457  (417, 497) | 472  (444, 501) | 502  (480, 524) | 499  (481, 516) | 1.63  (0.85, 2.41) | **<0.001*** |
| **Prior CVD** | | | | | | | |
| Male | Present | 521  (480, 562) | 560  (525, 595) | 575  (543, 607) | 625  (594, 656) | 3.46  (0.86, 6.13) | **0.009*** |
|  | Absent or Unknown | 380  (369, 390) | 390  (382, 398) | 396  (389, 404) | 380  (373, 387) | -0.19  (-0.77, 0.39) | 0.514 |
| Female | Present | 379  (344, 413) | 452  (420, 485) | 452  (422, 483) | 446  (417, 475) | 2.88  (1.53, 4.25) | **<0.001*** |
|  | Absent or Unknown | 264  (257, 271) | 265  (260, 271) | 273  (268, 278) | 250  (246, 255) | -0.55  (-1.56, 0.48) | 0.294 |
| **Ethnicity** | | | | | | | |
| Male | Bumiputera | 437  (423, 451) | 453  (442, 464) | 455  (446, 465) | 435  (426, 443) | -0.02  (-0.93, 0.90) | 0.973 |
|  | Chinese | 327  (308, 346) | 322  (308, 337) | 336  (322, 350) | 329  (316, 342) | -0.31  (-0.77, 0.16) | 0.198 |
|  | Indian | 357  (333, 382) | 376  (357, 395) | 373  (355, 390) | 354  (338, 370) | -0.87  (-1.79, 0.05) | 0.065 |
|  | Others | 294  (171, 417) | 429  (316, 542) | 367  (276, 459) | 316  (237, 394) | 0.99  (-4.47, 6.75) | 0.729 |
| Female | Bumiputera | 304  (295, 313) | 310  (303, 317) | 318  (311, 324) | 287  (282, 29) | -0.55  (-1.73, 0.65) | 0.370 |
|  | Chinese | 207  (193, 222) | 211  (200, 223) | 207  (197, 218) | 185  (176, 195) | -0.99  (-2.32, 0.37) | 0.153 |
|  | Indian | 255  (238, 272) | 244  (231, 257) | 243  (231, 255) | 227  (216, 238) | -1.86  (-3.39, -0.30) | **0.019*** |
|  | Others | 358  (244, 472) | 233  (163, 304) | 265  (199, 332) | 253  (194, 311) | 0.13  (-4.78, 5.29) | 0.960 |
| **Region** | | | | | | | |
| Male | Central | 363  (348, 378) | 370  (357, 382) | 374  (363, 386) | 357  (347, 367) | -0.70  (-1.34, -0.05) | **0.034*** |
|  | East Coast | 459  (432, 487) | 481  (459, 504) | 484  (464, 504) | 453  (435, 471) | -0.34  (-1.88, 1.23) | 0.670 |
|  | Northern | 410  (390, 431) | 409  (394, 424) | 414  (401, 428) | 409  (397, 421) | 0.26  (-0.33, 0.86) | 0.391 |
|  | Southern | 383  (357, 409) | 406  (387, 426) | 420  (402, 438) | 411  (395, 428) | 1.12  (0.36, 1.88) | **0.004*** |
| Female | Central | 252  (241, 262) | 250  (242, 259) | 257  (249, 265) | 224  (217, 232) | -0.87  (-2.47, 0.76) | 0.293 |
|  | East Coast | 336  (318, 355) | 334  (319, 349) | 336  (323, 349) | 315  (303, 327) | -1.02  (-1.78, -0.25) | **0.009*** |
|  | Northern | 276  (262, 290) | 285  (274, 295) | 287  (277, 296) | 265  (256, 273) | -0.27  (-1.48, 0.95) | 0.664 |
|  | Southern | 255  (238, 273) | 269  (255, 282) | 276  (263, 288) | 261  (250, 273) | -0.20  (-1.61, 1.22) | 0.779 |

Footnotes: All numbers in brackets are 95% confidence intervals of either the mortality rates or AAPC. Cardiovascular disease (CVD) was defined as the presence of either ischemic heart disease, stroke or amputation at registration into the registry. The Bumiputera ethnicity is a term used in Malaysia to describe Malays and other native people. AAPC, average annual percent change.

# **Table S7.** Trends in standardized mortality ratios in people aged 40-79 years with diagnosed type 2 diabetes in Malaysia, after excluding those who were diagnosed at younger than 30 years of age

|  | | **2012** | **2015** | **2017** | **2019** |
| --- | --- | --- | --- | --- | --- |
| **Overall** | | | | | |
| Male | | 1.75 (1.73, 1.79) | 1.82 (1.79, 1.85) | 1.92 (1.89, 1.95) | 1.82 (1.79, 1.85) |
| Female | | 1.78 (1.75, 1.81) | 1.88 (1.85, 1.91) | 2.01 (1.98, 2.05) | 1.84 (1.81, 1.88) |
| **Age group, years** | | | | | |
| Male | 40-49 | 3.36 (3.10, 3.63) | 3.92 (3.65, 4.21) | 4.26 (3.98, 4.57) | 3.75 (3.49, 4.03) |
|  | 50-59 | 2.32 (2.23, 2.41) | 2.54 (2.45, 2.64) | 2.75 (2.65, 2.85) | 2.58 (2.49, 2.68) |
|  | 60-69 | 1.84 (1.79, 1.89) | 1.85 (1.80, 1.90) | 1.95 (1.90, 2.00) | 1.82 (1.77, 1.87) |
|  | 70-79 | 1.43 (1.39, 1.47) | 1.43 (1.39, 1.47) | 1.48 (1.44, 1.52) | 1.43 (1.39, 1.47) |
| Female | 40-49 | 3.86 (3.53, 4.20) | 3.59 (3.28, 3.92) | 4.34 (4.00, 4.71) | 4.15 (3.82, 4.49) |
|  | 50-59 | 2.53 (2.42, 2.63) | 2.86 (2.74, 2.97) | 2.83 (2.72, 2.94) | 2.52 (2.41, 2.62) |
|  | 60-69 | 1.96 (1.90, 2.01) | 2.01 (1.96, 2.07) | 2.15 (2.09, 2.2) | 1.93 (1.87, 1.98) |
|  | 70-79 | 1.41 (1.38, 1.45) | 1.49 (1.45, 1.53) | 1.58 (1.54, 1.63) | 1.47 (1.43, 1.51) |
| **Duration of diabetes, years** | | | | | |
| Male | <5 | 1.67 (1.62, 1.73) | 1.67 (1.61, 1.73) | 1.71 (1.65, 1.77) | 1.58 (1.52, 1.64) |
|  | 5-10 | 1.73 (1.68, 1.78) | 1.80 (1.74, 1.85) | 1.90 (1.85, 1.96) | 1.78 (1.73, 1.84) |
|  | 10-15 | 1.80 (1.73, 1.86) | 1.96 (1.89, 2.03) | 2.06 (2.00, 2.13) | 1.92 (1.86, 1.99) |
|  | >15 | 1.90 (1.82, 1.98) | 1.91 (1.83, 2.00) | 2.09 (2.01, 2.18) | 2.15 (2.06, 2.24) |
| Female | <5 | 1.45 (1.39, 1.51) | 1.45 (1.39, 1.51) | 1.49 (1.43, 1.55) | 1.32 (1.26, 1.38) |
|  | 5-10 | 1.74 (1.69, 1.79) | 1.73 (1.68, 1.78) | 1.85 (1.79, 1.90) | 1.67 (1.62, 1.73) |
|  | 10-15 | 1.90 (1.84, 1.96) | 2.18 (2.12, 2.25) | 2.31 (2.24, 2.38) | 2.04 (1.97, 2.10) |
|  | >15 | 2.08 (2.01, 2.17) | 2.26 (2.18, 2.34) | 2.55 (2.46, 2.64) | 2.55 (2.46, 2.65) |
| **Prior CVD** | | | | | |
| Male | Present | 2.32 (2.29, 2.36) | 2.52 (2.49, 2.56) | 2.69 (2.65, 2.73) | 2.86 (2.82, 2.90) |
|  | Absent or Unknown | 1.69 (1.67, 1.72) | 1.76 (1.73, 1.79) | 1.85 (1.82, 1.89) | 1.74 (1.71, 1.77) |
| Female | Present | 2.48 (2.45, 2.52) | 3.08 (3.04, 3.13) | 3.23 (3.19, 3.27) | 3.19 (3.14, 3.23) |
|  | Absent or Unknown | 1.73 (1.70, 1.76) | 1.81 (1.78, 1.84) | 1.95 (1.92, 1.98) | 1.79 (1.76, 1.82) |
| **Ethnicity** | | | | | |
| Male | Bumiputera | 1.77 (1.74, 1.80) | 1.85 (1.82, 1.88) | 1.94 (1.91, 1.97) | 1.81 (1.78, 1.84) |
|  | Chinese | 1.78 (1.75, 1.81) | 1.78 (1.74, 1.81) | 1.92 (1.88, 1.95) | 1.86 (1.83, 1.90) |
|  | Indian | 1.30 (1.28, 1.32) | 1.39 (1.37, 1.42) | 1.46 (1.43, 1.48) | 1.34 (1.32, 1.37) |
|  | Others | 1.48 (1.45, 1.51) | 2.16 (2.13, 2.20) | 1.95 (1.92, 1.99) | 1.55 (1.52, 1.58) |
| Female | Bumiputera | 1.76 (1.73, 1.79) | 1.87 (1.84, 1.90) | 2.00 (1.97, 2.03) | 1.81 (1.78, 1.84) |
|  | Chinese | 1.83 (1.80, 1.87) | 1.95 (1.91, 1.99) | 2.02 (1.98, 2.06) | 1.87 (1.83, 1.91) |
|  | Indian | 1.53 (1.50, 1.56) | 1.50 (1.48, 1.53) | 1.59 (1.56, 1.62) | 1.41 (1.39, 1.44) |
|  | Others | 2.01 (1.98, 2.04) | 1.36 (1.34, 1.39) | 1.69 (1.66, 1.72) | 1.47 (1.44, 1.50) |

Footnotes: All numbers in brackets are 95% confidence intervals of standardized mortality ratios. Cardiovascular disease (CVD) was defined as the presence of either ischemic heart disease, stroke, or amputation at registration into the registry. The Bumiputera ethnicity is a term used in Malaysia to describe Malays and other native people.
